# CCR2/CCR5 inhibitor permits the radiation-induced effector T cell infiltration in pancreatic adenocarcinoma

**DOI:** 10.1084/jem.20211631

**Published:** 2022-04-11

**Authors:** Jianxin Wang, May Tun Saung, Keyu Li, Juan Fu, Kenji Fujiwara, Nan Niu, Stephen Muth, Junke Wang, Yao Xu, Noah Rozich, Haley Zlomke, Sophia Chen, Birginia Espinoza, MacKenzie Henderson, Vanessa Funes, Brian Herbst, Ding Ding, Christina Twyman-Saint Victor, Qihong Zhao, Amol Narang, Jin He, Lei Zheng

**Affiliations:** 1 Department of Oncology and the Sidney Kimmel Comprehensive Cancer Center, Johns Hopkins University School of Medicine, Baltimore, MD; 2 Department of Surgery, Johns Hopkins University School of Medicine, Baltimore, MD; 3 Department of Radiation Oncology, Johns Hopkins University School of Medicine, Baltimore, MD; 4 Pancreatic Cancer Precision Medicine Center of Excellence Program, Johns Hopkins University School of Medicine, Baltimore, MD; 5 The Bloomberg-Kimmel Institute for Cancer Immunotherapy, Johns Hopkins University School of Medicine, Baltimore, MD; 6 Bristol-Myers Squibb, Princeton, NJ

## Abstract

The resistance of pancreatic ductal adenocarcinoma (PDAC) to immune checkpoint inhibitors (ICIs) is attributed to the immune-quiescent and -suppressive tumor microenvironment (TME). We recently found that CCR2 and CCR5 were induced in PDAC following treatment with anti–PD-1 antibody (αPD-1); thus, we examined PDAC vaccine or radiation therapy (RT) as T cell priming mechanisms together with BMS-687681, a dual antagonist of CCR2 and CCR5 (CCR2/5i), in combination with αPD-1 as new treatment strategies. Using PDAC mouse models, we demonstrated that RT followed by αPD-1 and prolonged treatment with CCR2/5i conferred better antitumor efficacy than other combination treatments tested. The combination of RT + αPD-1 + CCR2/5i enhanced intratumoral effector and memory T cell infiltration but suppressed regulatory T cell, M2-like tumor–associated macrophage, and myeloid-derived suppressive cell infiltration. RNA sequencing showed that CCR2/5i partially inhibited RT-induced TLR2/4 and RAGE signaling, leading to decreased expression of immunosuppressive cytokines including CCL2/CCL5, but increased expression of effector T cell chemokines such as CCL17/CCL22. This study thus supports the clinical development of CCR2/5i in combination with RT and ICIs for PDAC treatment.

## Teaser

This preclinical study showed that CCR2/CCR5 dual-antagonist counteracts radiation-induced suppressive signals in myeloid cells and upregulates the effector T cell pathway and supported the ongoing clinical trial of the combination therapy of radiation, CCR2/CCR5 dual-antagonist, and anti–PD-1 antibody for locally advanced pancreatic cancer.

## Graphical Abstract

**Figure:**
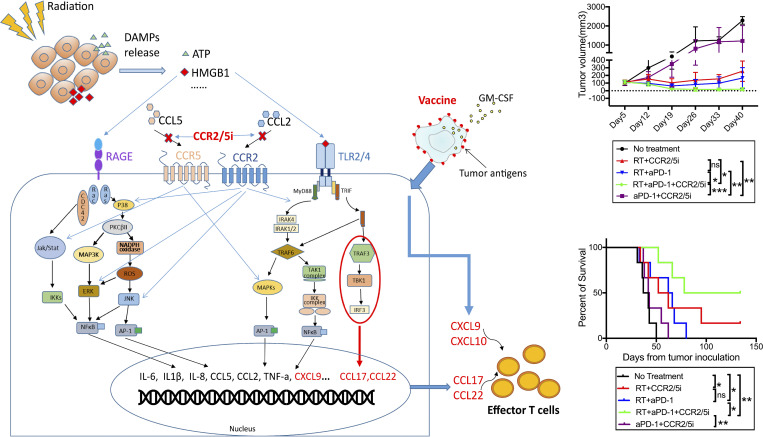

## Introduction

Pancreatic ductal adenocarcinoma (PDAC) has a dismal prognosis, with a 5-yr overall survival of 10% in patients of all stages (Kleeff et al., 2016). The duration of response to existing radiation and/or chemotherapy regimens is low in PDAC. Cancer immunotherapy, particularly the immune checkpoint inhibitor (ICI), has caused a paradigm shift in our treatment of cancer in the past decade (Galon and Bruni, 2019; Yang, 2015), but it has minimal clinical effect in PDAC (Morrison et al., 2018; Royal et al., 2010). The resistance of PDAC to ICIs is mainly attributed to the immune-quiescent, or “cold,” nature of the PDAC tumor microenvironment (TME).

Most of the tumor-infiltrating immune cells in PDAC are immunosuppressive cells, including regulatory T cells (Tregs; Wang et al., 2017; Yang, 2015), protumoral M2-like macrophages (Ma et al., 2016), and myeloid cells (Pushalkar et al., 2018), which impede the effects of cancers vaccines, T cell therapies, ICIs, or combinations thereof. One potential strategy is to target the Treg or immunosuppressive myeloid cells directly, but few agents are effective. Cabiralizumab, a monoclonal antibody targeting myeloid cells by inhibiting the CSF-1 receptor (CSF-1R), failed to confirm its benefit in patients with advanced PDAC in a phase 2 trial investigating cabiralizumab in combination with nivolumab, with or without chemotherapy (Five Prime Therapeutics, Inc., 2020). The possibilities underlying the failure of the cabiralizumab-based regimen include ineffective targeting of myeloid cells, lack of use of T cell–priming agents, and inadequate combinatorial effect from chemotherapy.

Whereas the CSF-1/CSF-1R axis is essential to the differentiation of myeloid cells including granulocytes, macrophages, and dendritic cells, the C-C motif chemokine receptor 2 and chemokine receptor 5 (CCR2 and CCR5) are important mediators of myeloid cell migration to nonhematopoietic organs and tissues including TME of pancreatic, colorectal, hepatocellular, and lung carcinomas.

CCR2 and its cognate ligand, CCL2, are implicated in the infiltration of immunosuppressive cells into tumors, notably M2-like tumor-associated macrophages (TAMs; Schmall et al., 2015) and myeloid-derived suppressor cells (MDSCs; Hartwig et al., 2017). Patients with PDAC tumors that exhibit high CCL2 expression and low CD8^+^ T cell infiltration have significantly lower survival (Lim et al., 2016). In mouse tumor models, CCR2 blockade depletes inflammatory resident monocytes and macrophages from the primary tumor and premetastatic liver, resulting in enhanced antitumor immunity, decreased tumor growth, and reduced metastasis (Sanford et al., 2013). Germline knockout of CCR2 or treatment with an anti-CCR2 antibody results in blockade of radiation-induced monocytic MDSC infiltration and enhances the antitumor effects of stimulator of interferon genes agonists and radiotherapy (RT; Liang et al., 2017). Tumor-derived CCL2 was shown to mediate resistance to radiation by recruiting CCR2^+^ monocytes in a mouse model of PDAC (Kalbasi et al., 2017). RNA microarray analysis of the microdissected lymphoid aggregates demonstrated that upregulation of CCL2 following treatment with a pancreatic cancer vaccine was associated with significantly poorer survival in patients with PDAC (Lutz et al., 2014), providing further evidence on the role of the CCL2/CCR2 axis in the cancer treatment response. A phase 1b trial targeting TAMs with a CCR2 inhibitor (PF-04136309) in combination with FOLFIRINOX in patients with stage III pancreatic cancer reported a promising, high objective tumor response rate. However, evidence on the role of targeting CCR2 in enhancing the efficacy of immunotherapy in PDAC or other cold tumors is still lacking.

CCR5 is another chemokine receptor that plays a role in the infiltration of both TAMs and Tregs into tumors (Nie et al., 2019). The CCR5 antagonist, Maraviroc, is a Food and Drug Administration–approved treatment for HIV with an already established safety profile (Woollard and Kanmogne, 2015). However, the role of CCR5 in PDAC is controversial. Published studies (Hundeyin et al., 2019; Mirlekar et al., 2020) suggest an immune-permissive role of CCR5 in PDAC. Nevertheless, in a mouse model of PDAC, knockdown of CCR5 from tumor cells resulted in suppression of tumor growth and significant decrease of Tregs in the TME, suggesting that the CCL5/CCR5 pathway has an impact on the infiltration of Tregs (Tan et al., 2009). Nevertheless, substantiated evidence of a direct role of CCL5/CCR5 on Tregs and TAMs in PDAC is still lacking.

It should be recognized that simply targeting CCR2 or CCR5 in PDAC may still fail to sensitize a cold tumor to ICIs in the absence of a T cell–priming mechanism. We previously reported that our GM-CSF–secreting allogeneic PDAC cell vaccine (GVAX) can induce the formation of tertiary lymphoid aggregates in PDACs within 2 wk following one vaccination, and that these aggregates serve as sites of T cell education to PDAC antigens (Lutz et al., 2014). Furthermore, GVAX induces PD-L1 expression on both the tumor epithelial cells and myeloid cells and causes PD-1^+^ T cell infiltration into these lymphoid aggregates, suggesting that vaccine therapy may prime PDACs to respond to ICIs (Lutz et al., 2014; Tsujikawa et al., 2017). Subsequently, we and others have shown that it is possible to turn the immunologic desert in PDACs into immune-responsive tumors (Tsujikawa et al., 2020). However, the response rates remain low in clinical trials investigating these regimens.

Our group thus has also examined other T cell–priming mechanisms. RT, one of conventional treatment modalities actively being used for PDAC, can cause immunogenic cell death, which activates innate responses including the receptor for advanced glycation endproducts (RAGE) and TLR2/4 pathways and subsequently modifies the TME by promoting the release of tumor antigens and chemokines that recruit inflammatory cells into the TME. To test the hypothesis that RT may serve as an “in situ vaccination,” we examined the combination of RT and ICIs in a mouse model of PDAC and found that this combination showed local antitumor efficacy but failed to induce effector T cell infiltration into the tumors (Fujiwara et al., 2020). Moreover, it is known that RT induces both antitumor immune cells, such as antigen-presenting cells that can potentially activate cytotoxic T cell function, as well as immunosuppressive cells such as MDSCs and TAMs (Jarosz-Biej et al., 2019).

Therefore, in this preclinical study, we examined GVAX and/or RT as T cell–priming mechanisms together with BMS-687681, a mouse surrogate for small-molecule dual antagonist of CCR2/CCR5, as an immunosuppressive TME-targeting agent, in combination with the anti–PD-1 ICI, as a new strategy for PDAC treatment.

## Results

### CCR2 and CCR5 expression are induced by treatment with GVAX and nivolumab and are associated with the immunosuppressive TME in human PDAC

**Figure 1.**
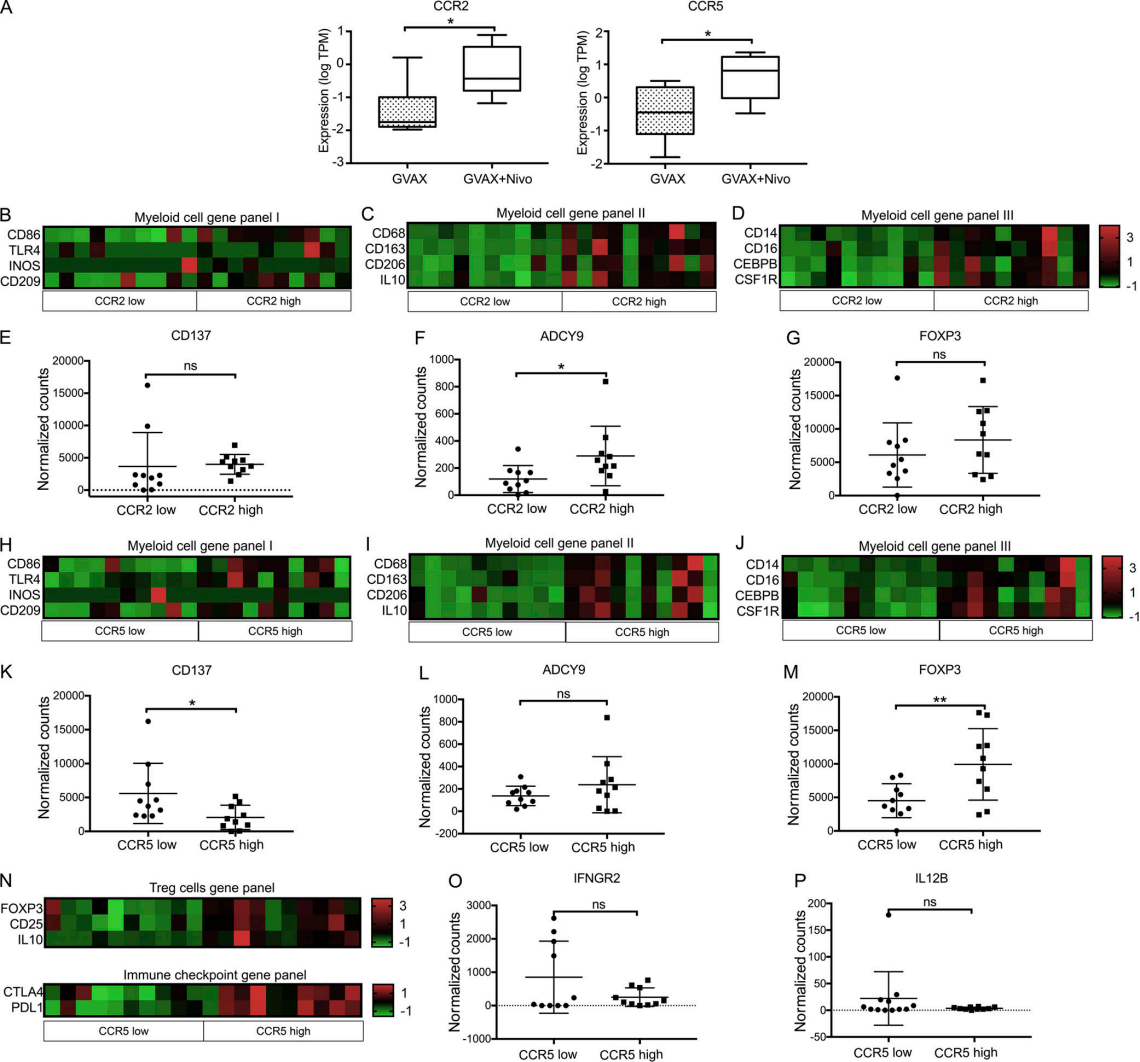
**RNAseq of isolated immune cells from human PDAC tissue.** Flow cytometry was used to sort CD4^+^, CD11b^+^, and CD8^+^ cells from immune cells isolated from PDAC resected from patients treated with GVAX or GVAX + nivolumab (nivo; *n* = 20). RNA was purified from sorted cell types, and RNAseq was performed. Normalized read count is shown. **(A)** Expression of *CCR2* and *CCR5* in sorted CD11b^+^ cells from PDACs belonging to the GVAX and GVAX + nivolumab treatment arms. The median expression level of CCR2 or CCR5 was used as the cutoff to divide the tumors into CCR2-low and CCR2-high subgroups or CCR5-low and CCR5-high subgroups. **(B–G)** For comparison between tumors with CCR2-low expression in CD11b^+^ cells vs. those with CCR2-high expression in CD11b^+^ cells, shown are heatmaps of gene expressions in CD11b^+^ cells of myeloid cell gene panel I, including the genes whose expression has been described in M1-like macrophages (B), myeloid cell gene panel II, including the genes whose expression has been described in M2-like macrophages (C), and myeloid cell gene panel III, including the genes whose expression has been described in MDSCs (D); shown are expression in CD8^+^ T cells of *CD137* (E) and *ADCY9* (F) and in CD4^+^ T cells of *FOXP3* (G). **(H–M)** For comparison between tumors with CCR5-low expression in CD11b^+^ cells vs. those with CCR5-high expression in CD11b^+^ cells, shown are heatmaps of gene expressions in CD11b^+^ cells of myeloid cell gene panel I, including the genes whose expression has been described in M1-like macrophages (H), myeloid cell gene panel II, including the genes whose expression has been described in M2-like macrophages (I), and myeloid cell gene panel III including the genes whose expression has been described in MDSCs (J); shown are expression in CD8^+^ T cells of *CD137* (K) and *ADCY9* (L) and in CD4^+^ T cells of *FOXP3* (M). **(N–P)** For comparison between tumors with CCR5-low expression in CD4^+^ T cells vs. those with CCR5-high expression in CD4^+^ T cells, shown are heatmaps of gene expressions in CD4^+^ T cells of Treg cell gene panel including the genes whose expression has been described in Tregs and immune checkpoint gene panel including *CTLA-4* and *PD-L1*; shown are gene expression in CD8^+^ T cells of *IFNGR2* (O) and *IL12B* (P). *, P < 0.05; **, P < 0.01, by unpaired *t* test.

**Figure S1.**
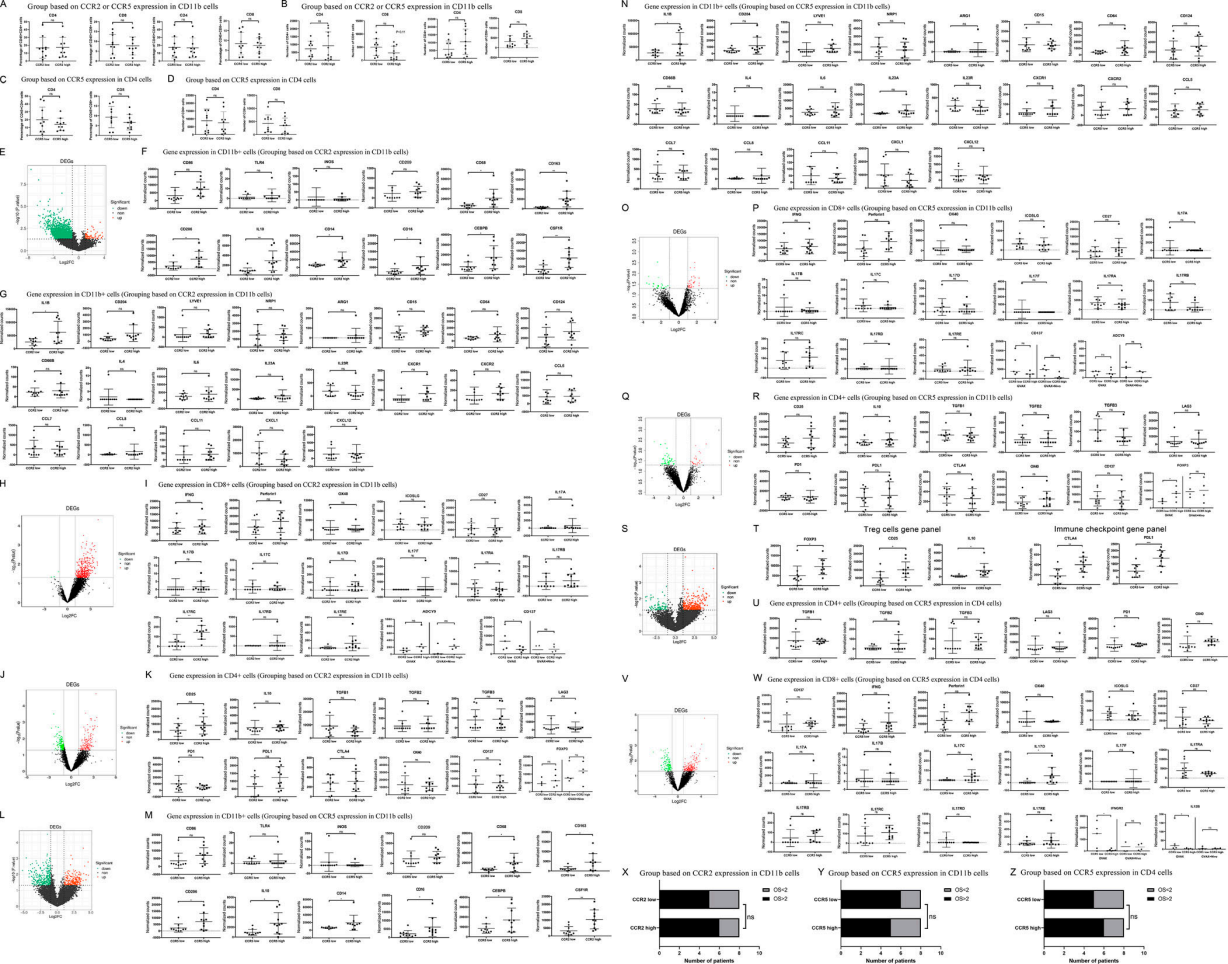
**Immunosuppressive cell infiltration was associated with CCR2 and CCR5 expression in human PDAC tissue.** Flow cytometry was used to sort CD4^+^, CD11b^+^, and CD8^+^ cells from immune cells isolated from PDACs resected from patients treated with GVAX or GVAX + nivolumab (nivo; *n* = 20). RNA was purified from sorted cell types, and RNAseq was performed. Normalized read count is shown. The median expression level of CCR2 or CCR5 was used as the cutoff to divide the tumors into CCR2-low and CCR2-high subgroups or CCR5-low and CCR5-high subgroups. **(A–D)** Shown are comparisons of percentages of CD4^+^ T cells or CD8^+^ T cells among CD45^+^ cells (A and C) or total numbers of CD4^+^ T cells or CD8^+^ T cells on flow cytometry when these cells were sorted (B and D). The samples in A and B were subgrouped based on CCR2 or CCR5 expression in CD11b^+^ cells; those in C and D were subgrouped based on CCR5 expression in CD4^+^ T cells. **(E)** Volcano plot for differential gene expression in CD11b^+^ cells with high vs. low CCR2 expression. **(F)** Expression of M1 macrophage-associated genes (*CD86*, *TLR4*, *iNOS*, and *CD209*) in CD11b^+^ cells with high vs. low CCR2 expression is shown; these genes were not significantly changed in CD11b^+^ cells with high vs. low CCR2 expression according to the volcano plot in E. Expression of M2 macrophage-associated genes (*CD68*, *CD163*, *CD206*, and *IL10*) and MDSC-associated genes (*CD14*, *CD16*, *CEBPB*, and *CSF1R*) in CD11b^+^ cells with high vs. low CCR2 expression is shown; these genes were among those significantly upregulated in CD11b^+^ cells with high CCR2 expression according to the volcano plot in E. **(G)** Tumors were subgrouped by CCR2 expression levels in CD11b^+^ cells, and expression of selected genes in CCR2^hi^ CD11b^+^ cells was compared to those in CCR2^lo^ CD11b^+^ cells. **(H)** Volcano plot for differential gene expression in CD8^+^ T cells when tumors were subgrouped by the CCR2 expression levels in CD11b^+^ cells. **(I)** Expression of selected genes in CD8^+^ T cells was compared between tumors with CCR2^hi^ CD11b^+^ cells vs. those with CCR2^lo^ CD11b^+^ cells. Expression of *CD137* and *ADCY9* was also compared within the GVAX and GVAX + Nivo treatment groups, respectively. **(J)** Volcano plot for differential gene expression in CD4^+^ T cells when tumors were subgrouped by CCR2 expression levels on CD11b^+^ cells. **(K)** Expression of selected genes in CD4^+^ T cells was compared between tumors with CCR2^hi^ CD11b^+^ cells vs. those with CCR2^lo^ CD11b^+^ cells. Expression of *Foxp3* was also compared within the GVAX and GVAX + Nivo treatment groups. **(L)** Volcano plot for differential gene expression in CD11b^+^ cells with high vs. low CCR5 expression. **(M)** Expression of M1 macrophage-associated genes (*CD86*, *TLR4*, *iNOS*, and *CD209*) in CD11b^+^ cells with high vs. low CCR5 expression is shown; these genes were not significantly changed in CD11b^+^ cells with high vs. low CCR5 expression according to the volcano plot in L. Expression of M2 macrophage-associated genes (*CD68*, *CD163*, *CD206*, and *IL10*) and MDSC-associated genes (*CD14*, *CD16*, *CEBPB*, and *CSF1R*) in CD11b^+^ cells with high vs. low CCR5 expression is shown; these genes were among those significantly upregulated in CD11b^+^ cells with high CCR5 expression according to the volcano plot in L. **(N)** Tumors were subgrouped by the CCR5 expression levels in CD11b^+^ cells, and expression of selected genes in CCR5^hi^ CD11b^+^ cells was compared to those in CCR5^lo^ CD11b^+^ cells. **(O)** Volcano plot for differential gene expression in CD8^+^ cells when tumors were subgrouped by CCR5 expression levels in CD11b^+^ cells. **(P)** Expression of selected genes in CD8^+^ T cells was compared between tumors with CCR5^hi^ CD11b^+^ cells vs. those with CCR5^lo^ CD11b^+^ cells. Expression of *CD137 *and *ADCY9* was also compared within the GVAX and GVAX + Nivo treatment groups, respectively. **(Q)** Volcano plot for differential gene expression in CD4^+^ T cells when tumors were subgrouped by CCR5 expression levels on CD11b^+^ cells. **(R)** Expression of select genes in CD4^+^ T cells was compared between tumors with CCR5^hi^ CD11b^+^ cells vs. those with CCR5^lo^ CD11b^+^ cells. Expression of *Foxp3* was also compared within the GVAX and GVAX + Nivo treatment groups. **(S)** Volcano plot for differential gene expression in CD4^+^ cells when tumors were subgrouped by CCR5 expression levels in CD4^+^ cells. **(T)** Expression of Treg cell markers (*FOXP3*, *CD25*, and *IL10*) and immune checkpoints (*CTLA4* and *PD-L1*) in CD4^+^ T cells with high vs. low CCR5 expression is shown; these genes were among those significantly upregulated in CD4^+^ T cells with high CCR5 expression according to the Volcano plot in S. **(U)** Expression of selected genes in CD4^+^ T cells was compared between tumors with CCR5^hi^ CD4^+^ T cells vs. those with CCR5^lo^ CD4^+^ T cells. **(V)** Volcano plot for differential gene expression in CD8^+^ T cells when tumors were subgrouped by CCR5 expression levels in CD4^+^ T cells. **(W)** Expression of selected genes in CD8^+^ T cells was compared between tumors with CCR5^hi^ CD4^+^ T cells vs. those with CCR5^lo^ CD4^+^ T cells. Expression of *IFNGR2* and *IL12B* was also compared within the GVAX and GVAX + Nivo treatment groups, respectively. *, P < 0.05; **, P < 0.01, by unpaired *t* test. **(X–Z)** Shown are the correlation analyses between numbers of tumors associated with overall survival (OS) >2 yr vs. OS <2 yr and subgroups based on CCR2 expression in CD11b^+^ cells (X), subgroups based on CCR5 expression in CD11b^+^ cells (Y), or subgroups based on CCR5 expression in CD4^+^ T cells (Z). χ^2^ test was used.

The deidentified analysis of the existing RNA sequencing (RNAseq) data from a recent clinical trial (NCT02451982) investigating neoadjuvant GVAX and nivolumab combination therapy demonstrated significantly greater CCR2 and CCR5 expression in CD11b^+^ myeloid cells (Fig. 1 A) sorted from PDAC specimens resected from patients who received GVAX + nivolumab compared with PDAC specimens resected from patients who were treated with GVAX alone (primary RNAseq result analysis and the clinical trial data not yet published). It is known that CCR2 and CCR5 played roles in the recruitment of bone marrow–derived monocytes and Tregs into peripheral tissues (Nie et al., 2019; Qian et al., 2011). Thus in this study, to understand such roles of CCR2 and CCR5 in PDAC, using the above deidentified, existing RNAseq data, we performed correlative analysis of the gene expression of markers of relevant immune cell types and the differential expression of *CCR2* and *CCR5* genes in CD11b^+^, CD4^+^, and CD8^+^ cells sorted from the tumor-infiltrating immune cells in the above PDAC specimens resected from patients who received GVAX or GVAX + nivolumab. The CCR2^hi^ (higher expression of CCR2) and CCR2^lo^ (lower expression of CCR2) subgroups or CCR5^hi^ and CCR5^lo^ subgroups of PDACs had similar percentages of CD4^+^ T cells or CD8^+^ T cells among CD45^+^ cells or total numbers of CD4^+^ T cells or CD8^+^ T cells on flow cytometry when these cells were sorted (Fig. S1, A–D). In these PDACs, higher CCR2 expression in CD11b^+^ cells was associated with significantly higher expression of genes that have been described in M2-like macrophages (*CD68/CD163/CD206/IL-10*) and MDSCs (*CD14/CD16/CEBPB/CSF-1R*; Fig. 1, B and D; and Fig. S1, E–G). Lower CCR2 expression in CD11b^+^ cells was associated with a trend toward higher costimulatory signal *CD137* expression and lower T cell suppressive signal *ADCY9* expression (Teixeira et al., 2017) in CD8^+^ cells (Fig. 1, E and F; and Fig. S1, H and I). In contrast, *FOXP3* expression in CD4^+^ cells was not affected by CCR2 expression in CD11b^+^ cells (Fig. 1 G and Fig. S1, J and K). These findings suggested that the presence of CCR2^hi^ CD11b^+^ myeloid cells might increase the density of M2-like macrophages and MDSCs and suppress effector T cell function in the PDACs, but it should be noted that M2 macrophages and MDSCs are not defined by transcriptomics.

Similar observations were noted when tumors were subgrouped by CCR5 expression in CD11b^+^ cells (Fig. 1, H–J; and Fig. S1, L–N). Expression of general macrophage-associated genes such as CSF-1R, but not M1-like macrophage-associated genes (*CD86/iNOS/TLR4/CD209*), was significantly different between tumors with CCR2^lo^ CD11b^+^ cells versus those with CCR2^hi^ CD11b^+^ cells and tumors with CCR5^lo^ versus CCR5^hi^ CD11b^+^ cells, suggesting that CCR2 and CCR5 upregulation possibly promotes either the macrophage polarization toward M2-like macrophages or the infiltration of M2-like macrophages (Fig. 1, C and I; and Fig. S1, F and M). Furthermore, tumors with CCR5^hi^ CD11b^+^ cells were associated with lower expression of *CD137* and a trend of higher expression of *ADCY9* in CD8^+^ cells (Fig. 1, K and L; and Fig. S1, O and P). Expression of *FOXP3* in CD4^+^ cells was increased in tumors with CCR5^hi^ CD11b^+^ cells (Fig. 1 M and Fig. S1, Q and R), suggesting that CCR5 in myeloid cells may also have a role in suppressing T cell activation through Tregs.

In addition, the expression of *Foxp3*, *CD25*, *IL10*, *CTLA-4*, and *PD-L1* genes increased in CCR5^hi^ CD4^+^ cells compared with CCR5^lo^ CD4^+^ cells, suggesting that CCR5 plays a role in the infiltration and/or function of Tregs in the TME (Fig. 1 N and Fig. S1, S and T). CCR5 expression level did not affect *PD-1 *and *LAG3* gene expression in CD4^+^ cells (Fig. S1 U). Consistently, expression of effector T cell cytokine/cytokine receptor genes such as *IFNGR2* and *IL12B* in CD8^+^ cells was decreased in tumors with CCR5^hi^ CD4^+^ cells following vaccine therapy (Fig. 1, O and P; and Fig. S1, V and W). Taken together, these results suggested that upregulation of CCR2 and CCR5 expression was likely associated with immunosuppressive TME and T cell–suppressive functions in patients with PDAC who received GVAX or GVAX + nivolumab and were potential targets for combination immunotherapy.

### Addition of GVAX to the combination of CCR2/5 dual-antagonist and anti–PD-1 antibody (αPD-1) does not lead to improved antitumor activity after RT treatment

**Figure S2.**
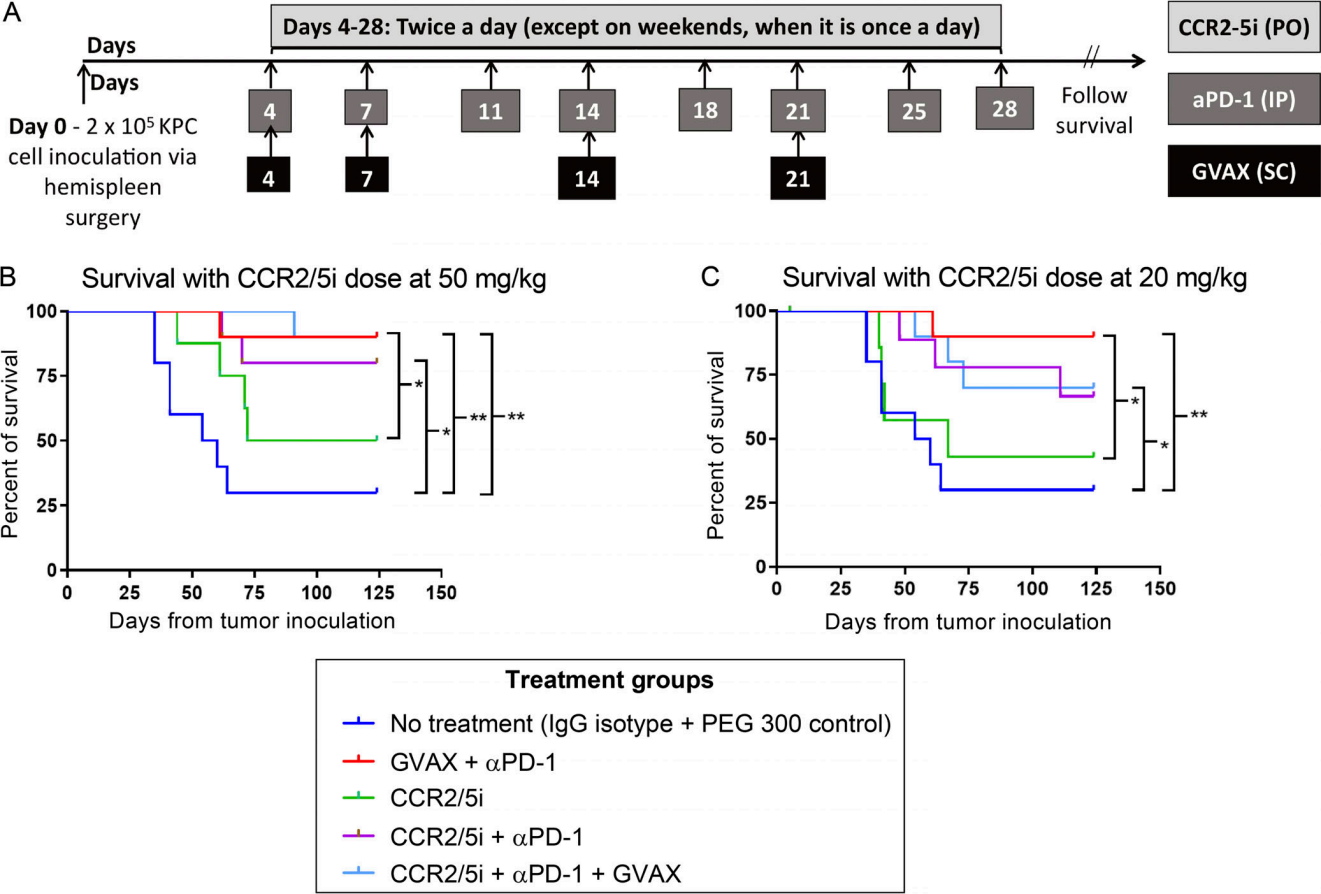
**Adding GVAX to dual-antagonism of CCR2 and CCR5 in combination with αPD-1 does not significantly enhance survival in a murine PDAC model.** To test the hypothesis that dual inhibition of CCR2 and CCR5 would enhance the antitumor activity of αPD-1 with or without the pancreatic cancer vaccine, GVAX, we used a syngeneic mouse model with diffuse liver metastases that were established by hemispleen injection of mouse KPC PDAC cells derived from KPC mice. Multiple preclinical studies of immunotherapy have used this mouse model because the TME in the liver resembles human PDACs, and the survival of the mice can be used to evaluate the antitumoral efficacy of the study treatments (Blair et al., 2019a; Blair et al., 2019b; Fujiwara et al., 2020; Kim et al., 2019; Soares et al., 2015a; Soares et al., 2015b). In this study, the mice with liver metastases were treated with a murine equivalent of GVAX, as previously described (Soares et al., 2015b), αPD-1, and a small-molecule dual antagonist of CCR2 and CCR5 (CCR2/5i, BMS-687681). BMS-687681 specifically binds to both CCR2 and CCR5 and subsequently inhibits the activation of CCR2/CCR5-mediated signal transduction pathways (Norman, 2011). **(A)** According to its pharmacodynamics (Norman, 2011), this CCR2/5i was tested at two different doses in this study. In this experiment, liver metastases were heterogeneous among the mice; thus, a small percentage of mice in the vehicle-treated control (no treatment) group remained alive at day 120 when the experiment was ended. **(B and C)** Single-agent CCR2/5i dosed at 20 mg/kg did not appear to confer any antitumor activity compared with the control group, whereas CCR2/5i dosed at 50 mg/kg as a single agent conferred modest antitumor activity. In addition, CCR2/5i dosed at 50 mg/kg did not result in any noticeable toxicity either as a single agent or in combination with other agents through the entirety of the study. Therefore, the 50 mg/kg dose of CCR2/5i was chosen for subsequent experiments. When CCR2/5i was combined with αPD-1, it significantly improved survival compared with the control group, but not significantly compared with CCR2/5i as a single agent. In this experiment, αPD-1 as a single agent was not examined, because this treatment had been tested multiple times in previously published studies and showed a single-agent activity as modest as that of CCR2/5i as a single agent (Muth et al., 2021; Soares et al., 2015b). Nevertheless, the combination of GVAX and αPD-1 showed significantly better antitumor activity than CCR2/5i as a single agent. However, adding CCR2/5i at 50 mg/kg to the combination of GVAX and αPD-1 did not lead to an improvement of survival (Fig. S2 B). The triple combination of αPD-1, CCR2/5i, and GVAX was only modestly better than the combination of αPD-1 and CCR2/5i, and this difference was not statistically significant, suggesting that GVAX may not be an adequate T cell priming agent in combination regimens that include both αPD-1 and CCR2/5i. **(A)** Schema of tumor implantation by the hemispleen procedure and treatment with GVAX, αPD-1, and CCR2/5i. Mice received 2 × 10^5^ KPC cells via the hemispleen procedure, followed by administration of GVAX on days 4, 7, 14, and 21. αPD-1 or IgG control (5 mg/kg) was administered by i.p. injection twice weekly for 4 wk. CCR2/5i (20 or 50 mg/kg) was administered by oral gavage twice a day starting on day 4 until day 28. Kaplan–Meier survival curves of mice treated with different combinations of GVAX, αPD-1, and CCR2/5i 20 mg/kg (B) or 50 mg/kg (C). Data for all figures represent results obtained from experiments with 10 mice per treatment group. *, P < 0.05; **, P < 0.01, by log-rank test.

**Figure S3.**
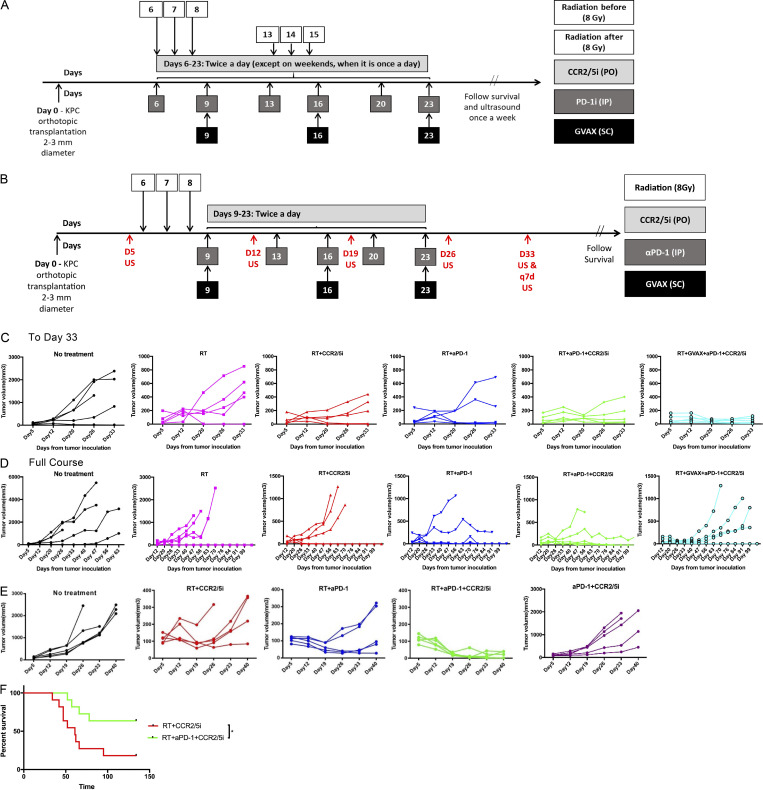
**The treatment schema and tumor growth curve in different treatment groups. (A)** Schema of orthotopic tumor implantation in mice, followed by treatment with GVAX, αPD-1 (5 mg/kg), CCR2/5i (50 mg/kg), and RT (3 fractions of 8 Gy). Two different schedules of RT were used (*n* = 5 per group). Ultrasound was used to monitor tumor size. Results are shown in Fig. 2, A and B. **(B)** Schema of orthotopic tumor implantation in mice, followed by treatment with GVAX, αPD-1 (5 mg/kg), CCR2/5i (50 mg/kg), and RT (3 fractions of 8 Gy). RT was administered prior to initiation of immunotherapy. Ultrasound was used to monitor tumor size. Results are shown in Fig. 2 C and below (C and D). **(C)** Tumor growth curves of different treatment groups as measured by ultrasound until day 33 from tumor implantation. **(D)** Tumor growth curves of different treatment groups as measured by ultrasound until death of mice or completion of experiment. **(E)** Experimental schema shown in Fig. 3 A. CCR2/5i was dosed twice daily continuously until death of mice or completion of experiment; tumor growth curves of different treatment groups were measured by ultrasound until day 33 from tumor implantation (*n* = 5 per group). **(F)** Kaplan–Meier survival curves of mice treated with different combinations. Data for all figures represent results obtained from experiments with 11 mice per treatment group. This analysis combines the results from two repeated experiment arms described in Fig. 3 A. *, P < 0.05, by log-rank test.

**Figure 2.**
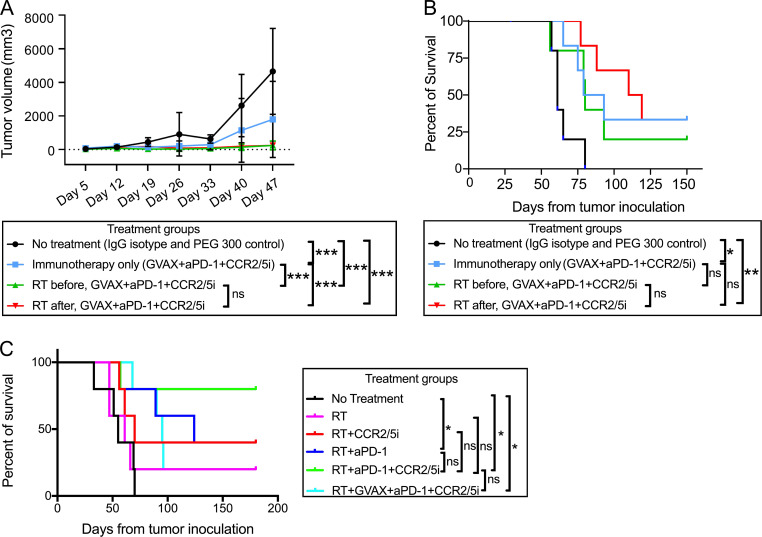
**The addition of RT further improved the antitumor activity of combination GVAX, αPD-1, and CCR2/5i therapy in a PDAC orthotopic mouse model. (A)** Tumor size evaluated with ultrasound imaging until day 47. **(B)** Kaplan–Meier survival curves of mice treated with two different sequences of RT administration relative to treatment with combination immunotherapy (GVAX + αPD-1 + CCR2/5i). **(C)** Kaplan–Meier survival curves of mice treated with different combinations of RT, GVAX, αPD-1, and CCR2/5i. Data represent results obtained from experiments with five to six mice per treatment group; all experiments were repeated twice. RT vs. RT + αPD-1 + CCR2/5i, P = 0.08. *, P < 0.05; **, P < 0.01; ***, P < 0.001, by log-rank test.

We found that the triple combination of αPD-1, a small-molecule dual antagonist of CCR2 and CCR5 (CCR2/5i, BMS-687681), and a murine equivalent of GVAX (Soares et al., 2015b) was only modestly better than the combination of αPD-1 and CCR2/5i in a PDAC hemispleen liver metastasis murine model, and this difference was not statistically significant (Fig. S2), suggesting that GVAX may not be an adequate T cell–priming agent in combination regimens that include both αPD-1 and CCR2/5i. It is also possible that the effect of CCR2/5i would be difficult to discern after the combination of GVAX and αPD-1 has already improved the survival of mice substantially. Therefore, we further explored whether the addition of RT to the triple combination of CCR2/5i + αPD-1 + GVAX could effectively slow tumor growth in a PDAC orthotopic murine model, which better resembles human PDAC. As described previously (Fujiwara et al., 2020), clips were placed around the implanted tumors to guide future treatment with stereotactic body radiation (SBRT). As shown in Fig. S3 A, 6 d after tumor implantation, the mice were treated with weekly GVAX, twice weekly αPD-1, and twice daily CCR2/5i for one 3-wk cycle. Two different schedules of SBRT were studied to investigate the impact of the sequence of RT and GVAX on antitumor efficacy, while keeping the schedule of other treatments unchanged. One group of mice received three daily doses of SBRT at 8 Gy on days 6–8, where the first treatment with SBRT was performed before administration of the first weekly GVAX dose on day 6 (designated “RT before”); another group of mice received three daily doses of SBRT at 8 Gy on days 13–15 (designated “RT after”). A small animal ultrasound was used to monitor the primary PDAC tumor growth. The results demonstrated that the triple-combination immunotherapy of CCR2/5i + αPD-1 + GVAX slowed the rate of tumor growth significantly compared with the control (no treatment) group, and that the addition of SBRT further significantly enhanced the antitumor effect of the triple immunotherapy regimen (Fig. 2 A). Most of the mice eventually died from systemic metastases, which were commonly seen in the liver and peritoneum and rarely observed in the lung. There was a trend toward a survival advantage when SBRT was added to the triple immunotherapy of CCR2/5i + αPD-1 + GVAX, but it was not statistically significant (Fig. 2 B). Therefore, our results suggested that adding SBRT improved the local tumor control of triple immunotherapy CCR2/5i + αPD-1 + GVAX, regardless of whether SBRT was administrated early or late in the immunotherapy treatment course. In addition, no statistically significant difference in tumor growth or survival was observed between SBRT before CCR2/5i + αPD-1 + GVAX and SBRT after CCR2/5i + αPD-1 + GVAX. Nevertheless, other modifications to the treatment sequence that we did not investigate may still impact tumor growth and mice survival.

We then repeated the experiment by testing SBRT in combination with different immunotherapy strategies, including αPD-1 alone, CCR2/5i alone, dual immunotherapy combination of αPD-1 + CCR2/5i, and triple immunotherapy combination of GVAX + αPD-1 + CCR2/5i. The treatment schema (Fig. S3 B) was slightly different from the above experiment. Here, SBRT (8 Gy once daily for 3 d) was given prior to all other treatments. SBRT alone demonstrated modest antitumor activity. SBRT followed by αPD-1 alone, CCR2/5i alone, or αPD-1 + CCR2/5i suppressed tumor growth in only some of the mice, and SBRT followed by αPD-1 + CCR2/5i + GVAX suppressed tumor growth in all the mice initially up to approximately day 33 (i.e., 33 d from orthotopic tumor implantation; Fig. S3 C). However, later in the course of ultrasound observation, most mice in the groups treated with SBRT followed by CCR2/5i alone or αPD-1 + CCR2/5i + GVAX had uncontrolled tumor growth (Fig. S3 D). SBRT followed by αPD-1 alone continued to maintain tumor growth control later in the course of ultrasound observation similar to early in the course for most of the mice. Furthermore, SBRT followed by αPD-1 + CCR2/5i also maintained tumor growth control in most of the mice during the later course of ultrasound observation, perhaps to a greater degree than SBRT followed by αPD-1 alone (Fig. S3, D and E). These results suggested that addition of CCR2/5i to RT and αPD-1 conferred a more durable response in the primary PDAC tumor. It should be noted that, although addition of GVAX to RT + αPD-1 + CCR2/5i conferred early tumor control, tumor growth appeared to accelerate later in the disease course. SBRT followed by αPD-1 + CCR2/5i consistently led to better survival compared to all other treatment groups (Fig. 2 C) and was significantly better than the untreated control group. Although SBRT followed by αPD-1 alone or by αPD-1 + CCR2/5i + GVAX led to a significantly better median survival than the control group, fewer mice in these two groups remained alive compared with the group that received SBRT followed by αPD-1 + CCR2/5i (Fig. 2 C). These findings suggested that CCR2/5i and αPD-1 together might modulate RT-exposed TME in such a way that does not require the addition of GVAX as a T cell–priming mechanism. Taken together, we decided to further investigate SBRT followed by αPD-1 + CCR2/5i.

### RT followed by αPD-1 treatment and a prolonged treatment course of CCR2/5 dual-antagonist confers superior antitumor response and survival to other treatment combinations in a mouse PDAC model

**Figure 3.**
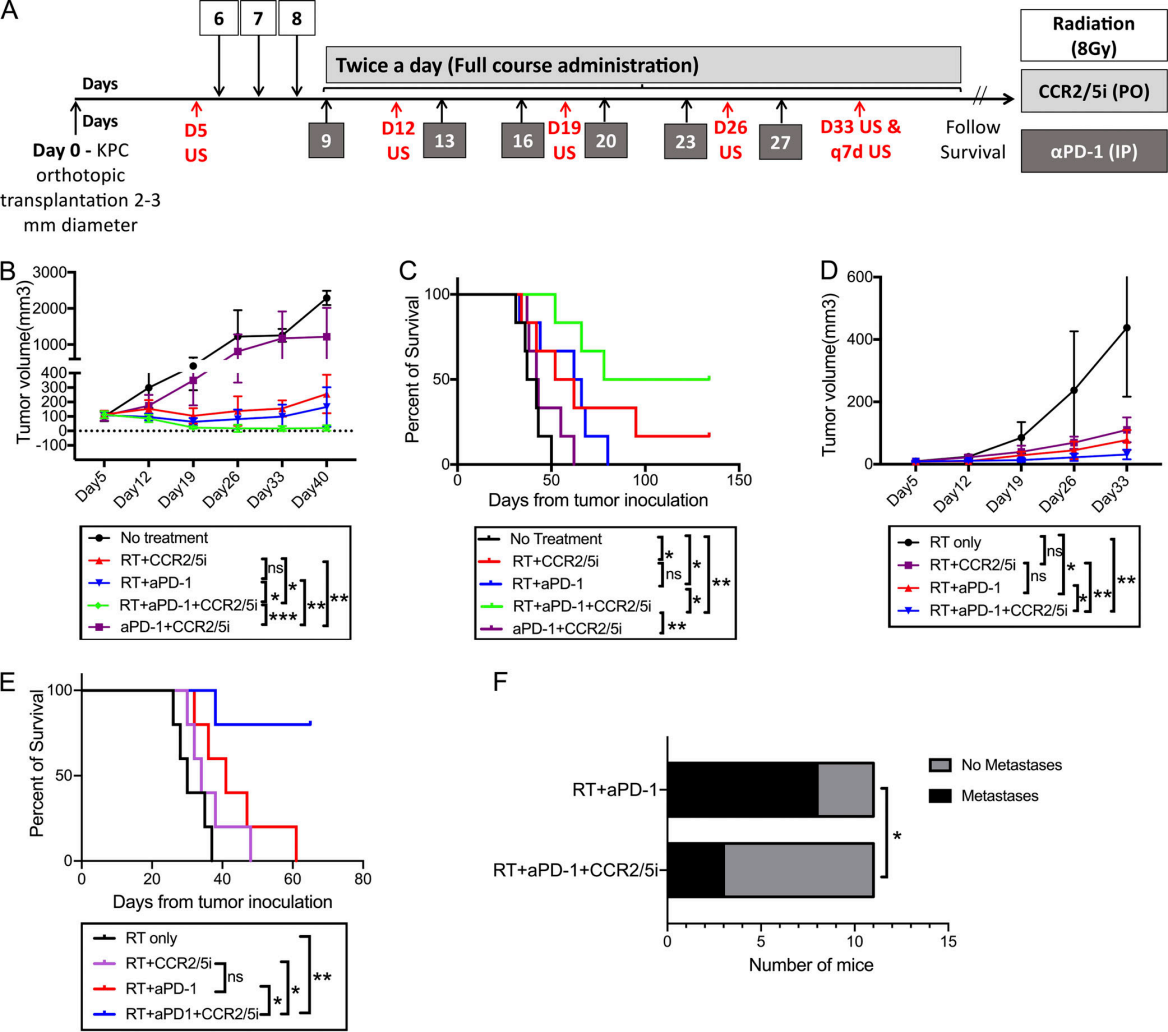
**The addition of CCR2/5i to RT and αPD-1 slowed the rate of tumor growth and prolonged survival in a PDAC orthotopic mouse model. (A)** Treatment schema: On day 0, subcutaneous tumors formed by injecting the KPC tumor cells onto syngeneic wild-type C57Bl/6 mice ∼1–2 weeks before were dissected and divided into cubes of 2–3-mm diameter. One cube of tumor was immediately implanted orthotopically into the pancreas of each syngeneic wild-type C57Bl/6 mouse. After the surgery, mice were randomized into different treatment groups (six mice per group) as indicated. On day 5 (D5), pretreatment ultrasound was performed. Tumor-bearing mice were treated with RT (three fractions of 8 Gy daily on days 6–8), αPD-1, or IgG control (5 mg/kg i.p. twice weekly for 3 wk), and CCR2/5i (50 mg/kg by oral gavage twice a day continuously) on days indicated. Ultrasound was performed on days indicated. **(B and C)** Tumor size evaluated by ultrasound imaging until day 40 (B) and Kaplan–Meier survival curves in mice treated with different combinations of RT, αPD-1, and CCR2/5i (C). **(D and E)** The same experiment was repeated in the orthotopic mouse model with a different mouse PDAC cell line established from KPC mice. After tumor implantation, mice were randomized into four treatment groups (*n* = 5 per group) as indicated. Tumor size evaluated by ultrasound imaging until day 33 (D) and Kaplan–Meier survival curves in mice treated with different combinations of RT, αPD-1, and CCR2/5i (E). **(F)** Comparison of metastases between RT + aPD1 + CCR2/5i and RT + aPD1 groups combining the experiment in B and C and one repeated experiment (*n* = 5 per group), in total 11 mice per group. After the mice reached survival endpoint (day 140), at necropsy, numbers of mice with lung, liver, or peritoneal metastases were identified grossly and histologically. Surviving mice were free of tumors. χ^2^ test was used to examine the correlation between treatment groups and metastasis rates. In the experiment in D and E, when the mice reached survival endpoint (day 63), all four surviving mice in the RT + aPD1 + CCR2/5i group were free of tumors; and the remaining one in the group did not have metastasis; all mice in the RT + aPD1 group had liver metastasis. *, P < 0.05; **, P < 0.01; ***, P < 0.001, by log-rank test. All experiments were repeated at least twice.

After the above experiments (Fig. 2), we investigated the contribution of treatment effect of each component of the SBRT followed by αPD-1 + CCR2/5i regimen, and also whether longer treatment with CCR2/5i in these combination regimens (i.e., treatment beyond day 23 until each mouse reached the survival endpoint) would confer better systemic disease control (Fig. 3 A). To this end, we conducted another experiment with Kras and p53 mutations conditional knock-in (KPC) tumors orthotopically implanted into mice and divided the mice into the following five treatment groups: no treatment, αPD-1 + CCR2/5i, SBRT followed by CCR2/5i, SBRT followed by αPD-1, and SBRT followed by αPD-1 + CCR2/5i. As in the above experiment, SBRT followed by αPD-1 + CCR2/5i conferred a significantly better control of primary pancreatic tumor growth compared with the other treatments tested (Figs. 3 B and S3 E). In addition, the SBRT + αPD-1 + CCR2/5i treatment group had significantly better survival than the SBRT + αPD-1 group (Fig. 3 C). As anticipated, the SBRT + αPD-1 + CCR2/5i treatment group had significantly better survival than the αPD-1 + CCR2/5i group. As in the above experiment (Fig. 2), this experiment demonstrated moderately prolonged survival with SBRT followed by CCR2/5i alone as the systemic treatment. We repeated the same experiment and confirmed that the SBRT + αPD-1 + CCR2/5i treatment was indeed superior to the SBRT + CCR2/5i treatment (Fig. S3 F). To further confirm the above results, we used another mouse PDAC tumor cell line, KPC4545, which was derived from the primary tumor of a KPC mouse with liver metastasis. This tumor cell line has a strong potential to develop liver metastases when it is orthotopically implanted in the pancreas (Pan et al., 2021). The SBRT + αPD-1 + CCR2/5i treatment group demonstrated significantly better local tumor control, better metastasis control, and improved survival in this model vs. any other treatment group including the SBRT + αPD-1 and SBRT + CCR2/5i treatment groups (Fig. 3, D and E). Necropsy of mice revealed a significantly higher metastasis rate in the SBRT + αPD-1 group than the SBRT + αPD-1 + CCR2/5i group, supporting our hypothesis that a longer course of CCR2/5i treatment improves systemic antitumor and antimetastasis activities (Fig. 3 F). Therefore, we concluded that SBRT followed by αPD-1 treatment and a prolonged treatment course of CCR2/5i was the best treatment strategy among all therapies that had been tested thus far for this mouse model of orthotopically implanted KPC tumor.

### The combination of CCR2/5 dual-antagonist, RT, and αPD-1 enhanced intratumoral effector and memory T cell infiltration

**Figure S4.**
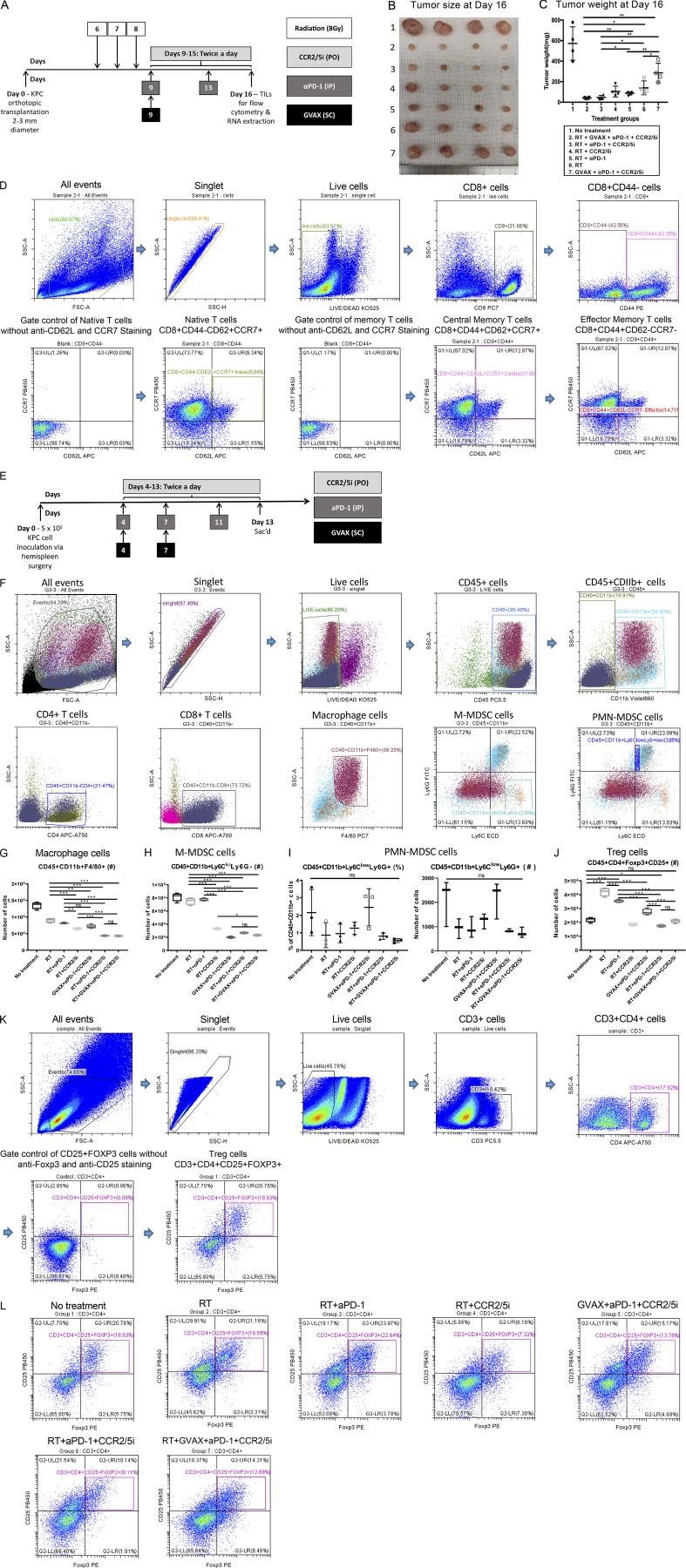
**CCR2/5 dual-antagonist in combination with RT and anti–PD-1 therapy enhanced the effector memory T cell infiltration and reversed the suppressive immune cell environment. (A)** Treatment schema of mice (*n* = 4 per group). Mice underwent orthotopic implantation and were treated with different combinations of GVAX, CCR2/5i (50 mg/kg), αPD-1 (5 mg/kg), and RT (3 fractions of 8 Gy on days 6–8). **(B and C)** Tumors were harvested at day 16 from tumor implantation (B) and the tumors were weighed (C). **(D)** Flow cytometry gating strategy for identification of naive/central memory/effector memory T cells. First, side scatter height (SSC-H) and side scatter area (SSC-A) plots were used to exclude doublets. Dead cells were excluded by gating on cells negative for the viability marker Aqua Blue. The expression of CD44 was used to identify naive (CD8^+^CD44^−^) and memory (CD8^+^CD44^+^) T cells. The naive T cells were defined as CD8^+^CD44^−^CD62^+^CCR7^+^. The expression of CD62L and CCR7 was used to define central memory T cells (CD8^+^CD44^+^CD62^+^CCR7^+^) and effector memory T cells (CD8^+^CD44^+^CD62^−^CCR7^−^). **(E)** Treatment schema of mice (*n* = 4 per group). Syngeneic mice underwent hemispleen surgery were treated with different combinations of GVAX, CCR2/5i (50 mg/kg), and αPD-1 (5 mg/kg). The mice were sacrificed on day 13 from hemispleen surgery, and the livers were harvested for IFN-γ ELISA. **(F)** Mice underwent orthotopic surgery and were treated with different combinations of GVAX, CCR2/5i (50 mg/kg), and αPD-1 (5 mg/kg). Mice were sacrificed on day 16 from orthotopic tumor implantation, and flow cytometry analysis was performed on the isolated tumor-infiltrating immune cells. The flow cytometry gating strategy of different cell types is shown. **(G)** Number of macrophages (CD45^+^CD11b^+^F4/80^+^) on flow cytometry analysis of immune cells isolated from orthotopically implanted KPC tumor resected from mice following treatment. The number of isolated tumor-infiltrating immune cells was normalized to the tumor weight. **(H)** Number of M-MDSCs (CD45^+^CD11b^+^Ly6C^hi^Ly6G^−^) on flow cytometry analysis of immune cells isolated from orthotopically implanted KPC tumor resected from mice following treatments as indicated. **(I)** Percentage within the total myeloid CD45^+^CD11b^+^ population and number of PMN-MDSCs (CD45^+^CD11b^+^Ly6C^low^Ly6G^+^) on flow cytometry analysis. **(J)** Number of Treg cells (CD45^+^CD11b^−^CD25^+^Foxp3^+^) on flow cytometry analysis of immune cells isolated from orthotopically implanted KPC tumor resected from mice following treatments as indicated. Data represent mean ± SEM from one representative experiment of four to five mice per treatment group, and the isolated immune cells from mice from the same treatment group were pooled and measured in triplicate. These experiments were repeated twice. *, P < 0.05; **, P < 0.01; ***, P < 0.001, by one-way ANOVA. **(K)** The flow cytometry gating strategy for Treg cells is shown. Lymphocytes were identified based on their forward- and side-scatter properties. Subsequently, singlet cells were gated; and dead cells were excluded by gating on cells negative for the viability marker Aqua Blue. CD3 and CD4 were used to identify T helper cells (CD3^+^CD4^+^) among the selected viable lymphocytes. Conventional Tregs were defined as CD4^+^ T cells coexpressing CD25 and FOXP3. **(L)** Shown are representative flow cytometry graphs of Treg cells (CD3^+^CD4^+^CD25^+^Foxp3^+^) in each treatment group as indicated.

**Figure 4.**
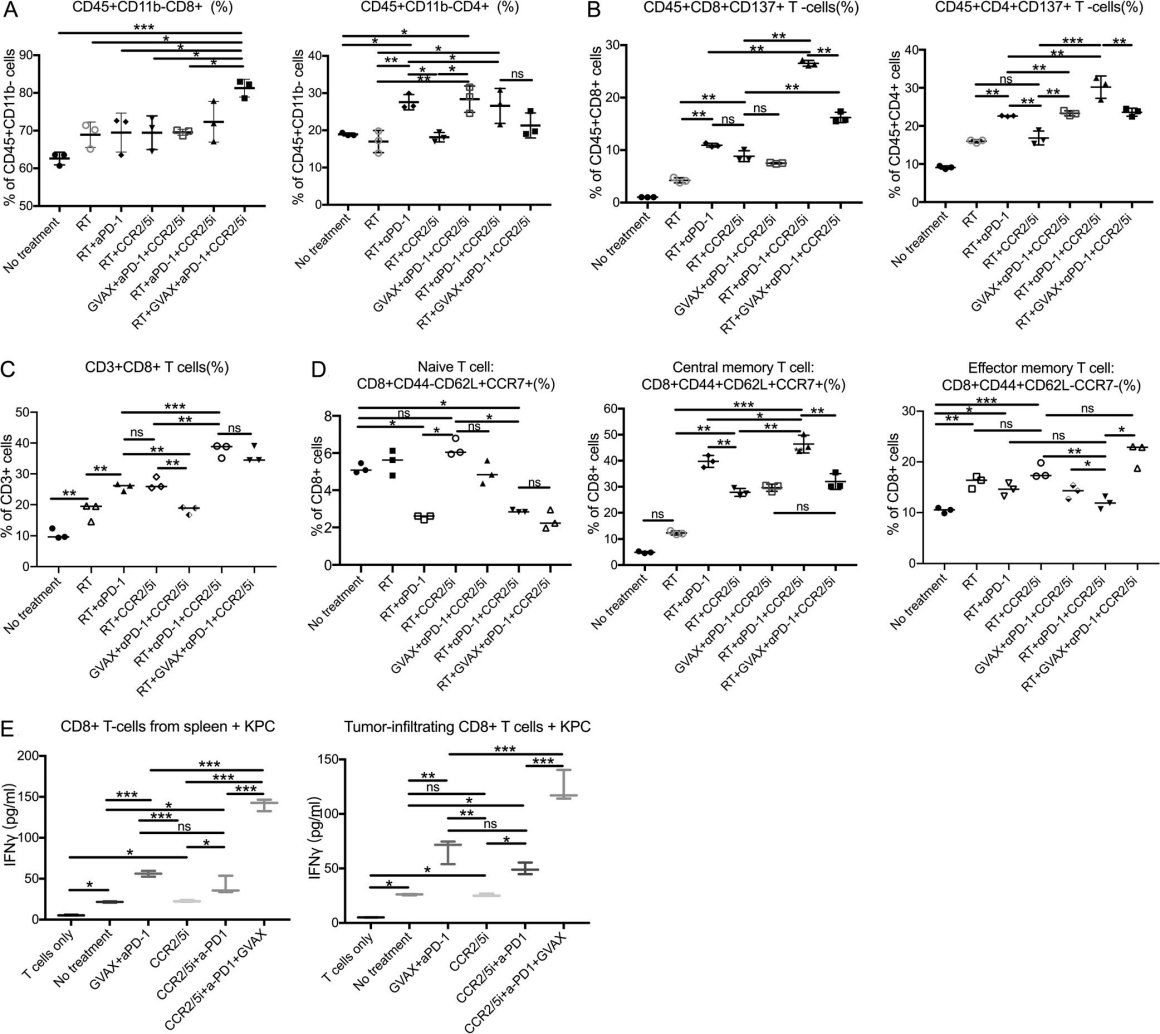
**CCR2/5 inhibitor in combination with RT and αPD-1 promoted T cell function in a PDAC orthotopic mouse model. (A–D)** Flow cytometry was performed on isolated tumor-infiltrating immune cells from dissected orthotopic tumor on day 16 (data in A and B were from one experiment, and data in C and D were from a separate experiment; *n* = 5 per group). The number of isolated tumor-infiltrating immune cells was normalized to the tumor weight, and the following were analyzed: percentage of CD8^+^ and CD4^+^ cells among CD45^+^ cells (A), CD137^+^ cells among CD45^+^CD8^+^ and CD45^+^CD4^+^ T cells (B), CD8^+^ cells among CD3^+^ cells (C), and naive T cell (CD8^+^CD44^−^CD62L^+^CCR7^+^), central memory T cells (CD8^+^CD44^+^CD62L^+^CCR7^+^), and effector memory T cells (CD8^+^CD44^+^CCR7^−^CD62L^−^) among CD8^+^ T cells (D). **(E)** CD8^+^ T cells were isolated and purified from the liver and spleen on day 13 after hemispleen injection of KPC cells into mice (*n* = 4 per group). ELISA assays were performed, using autologous irradiated KPC tumor cells as antigenic targets for CD8^+^ T cells isolated from the hepatic metastases and spleen. Data represent mean ± SEM from one representative experiment of four to five mice per treatment group, and the isolated CD8^+^ T cells from mice from the same treatment group were pooled and measured in triplicate. *, P < 0.05; **, P < 0.01; ***, P < 0.001, by one-way ANOVA.

To explore the immune mechanism of the CCR2/5i-based combination immunotherapy that led to the enhanced antitumor effect, we conducted another experiment in which we treated orthotopically implanted PDAC mice with short-course RT + immunotherapy as described in Fig. S4 A. We harvested the tumor-infiltrating immune cells on day 16 following tumor implantation for the flow cytometry analysis. Day 16 was chosen because the changes in the immune cells are more likely due to the treatments administered than an immune response to the tumor. Nevertheless, the volume and weight of the tumors from the RT + αPD-1 + CCR2/5i and RT + GVAX + αPD-1 + CCR2/5i groups were significantly smaller than those of the RT + αPD-1 and the RT + CCR2/5i groups (Fig. S4, B and C). Therefore, the quantification of tumor-infiltrating immune cells was normalized to the tumor weights. The results demonstrated that the percentage of CD8^+^ T cells among CD45^+^ cells increased in any treatment group (Fig. 4 A) compared with the untreated control group, although the increase was statistically significant only for the RT + GVAX + αPD-1 + CCR2/5i group. In contrast, the increase in percentage of CD4^+^ T cells among CD45^+^ cells was statistically significant in both the RT + GVAX + αPD-1 + CCR2/5i group and the RT + αPD-1 group compared with the untreated group. Interestingly, the intratumoral CD45^+^CD8^+^CD137^+^ and CD45^+^CD4^+^CD137^+^ activated T cells were significantly more numerous in the tumors treated with RT + αPD-1 + CCR2/5i than those treated with RT + αPD-1, RT + CCR2/5i, or any other treatment (Fig. 4 B). The addition of GVAX to RT + αPD-1 + CCR2/5i did not increase, but instead decreased, the percentage of CD8^+^CD137^+^ and CD4^+^CD137^+^ cells among CD8^+^ or CD4^+^ T cells, respectively (Fig. 4 B). These results suggested that RT in combination with both αPD-1 and CCR2/5i led to the activation of T cells in PDACs.

We next assessed the memory T cells in the tumors in a separate experiment using the same orthotopic KPC tumor implantation model. In this experiment, we observed a trend for the percentages of CD8^+^ T cells among CD3^+^ T cells (CD45 was not stained) in the tumors among the treatment groups (Fig. 4 C) similar to the percentages of CD8^+^ T cells among CD45^+^ cells (Fig. 4 A). The only exception is that the percentage of CD8^+^ T cells among CD3^+^ cells was higher than that of CD8^+^ T cells among CD45^+^ cells. This difference could result from a non–T cell component within CD45^+^ cells. We also observed an increase of the percentages of CD8^+^ T cells among CD3^+^ T cells in all treatment groups compared with the untreated group (Fig. 4 C). Compared with RT alone, RT + αPD-1, and RT + CCR2/5i treatment groups, the RT + αPD-1 + CCR2/5i and RT + GVAX + αPD-1 + CCR2/5i treatment groups significantly further increased the percentage of CD8^+^ T cells among CD3^+^ T cells (Fig. 4 C). As shown in Figs. 4 D and S4 D, the percentage of CD8^+^ naive T cells (CD8^+^CD44^−^CD62L^+^CCR7^−^) among intratumoral CD8^+^ T cells was significantly decreased in the RT + αPD-1 + CCR2/5i, RT + GVAX + αPD-1 + CCR2/5i, and RT + αPD-1 treatment groups. However, the percentage of central memory T cells (CD8^+^CD44^+^CD62L^+^CCR7^+^) was significantly increased in the RT + αPD-1 + CCR2/5i treatment group compared with any other treatment group. The percentage of central memory T cells was significantly increased in the RT + GVAX + αPD-1 + CCR2/5i group compared with the untreated group and the RT-only group, but not other treatment groups. In contrast, the percentage of effector memory T cells (CD8^+^CD44^+^CD62L^−^CCR7^−^) among CD8^+^ T cells was significantly increased in the RT + GVAX + αPD-1 + CCR2/5i group compared with all other groups except the RT + CCR2/5i group. However, the percentage of effector memory T cells among CD8^+^ T cells was not significantly increased the RT + αPD-1 + CCR2/5i treatment group as compared with any other treatment group and was even significantly lower than that in the RT + CCR2/5i treatment group. Such a result suggests that the main driver for the effector memory T cell infiltration is the RT + CCR2/5i treatment. αPD-1 may cause a decrease in effector memory T cells; however, considering the RT + αPD-1 + CCR2/5i treatment leads to a high intratumoral density of CD8^+^ cells, the overall density of effector memory T cells would still be high in this treatment group (Fig. 4 D).

To further determine whether CCR2/5i enhanced the function of infiltrating CD8^+^ T cells, we used the hemispleen metastatic liver mouse model to examine tumor-specific activity of systemic CD8^+^ T cells (isolated from the spleen) and tumor-infiltrating CD8^+^ T cells (isolated from liver metastases) using IFN-γ ELISA analysis with irradiated autologous KPC cells as the target (Fig. S4 E). As shown in Fig. 4 E, CCR2/5i alone did not increase IFN-γ secretion by CD8^+^ T cells compared with the control (no treatment) group. However, the GVAX + αPD-1 and CCR2/5i + αPD-1 treatment groups significantly enhanced IFN-γ production from CD8^+^ T cells isolated from the tumor and spleen compared with CCR2/5i alone. There was further increase in IFN-γ secretion from these isolated CD8^+^ T cells in the CCR2/5i + αPD-1 + GVAX group compared with either GVAX + αPD-1 or CCR2/5i + αPD-1 groups. These results suggested that CCR2/5i, αPD-1, and GVAX had synergistic effects on increasing the IFN-γ–mediated cytotoxic activity of T cells. Unfortunately, due to the small number of isolated CD8^+^ T cells, this assay was not sensitive enough to evaluate the IFN-γ–mediated cytotoxic activity of T cells in the orthotopically implanted pancreatic tumors following the RT treatment.

### CCR2/5 dual-antagonist in combination with RT and αPD-1 suppresses Tregs, M2-like TAMs, and monocytic (M)-MDSCs, but not M1-like TAMs and polymorphonuclear (PMN)-MDSCs

**Figure 5.**
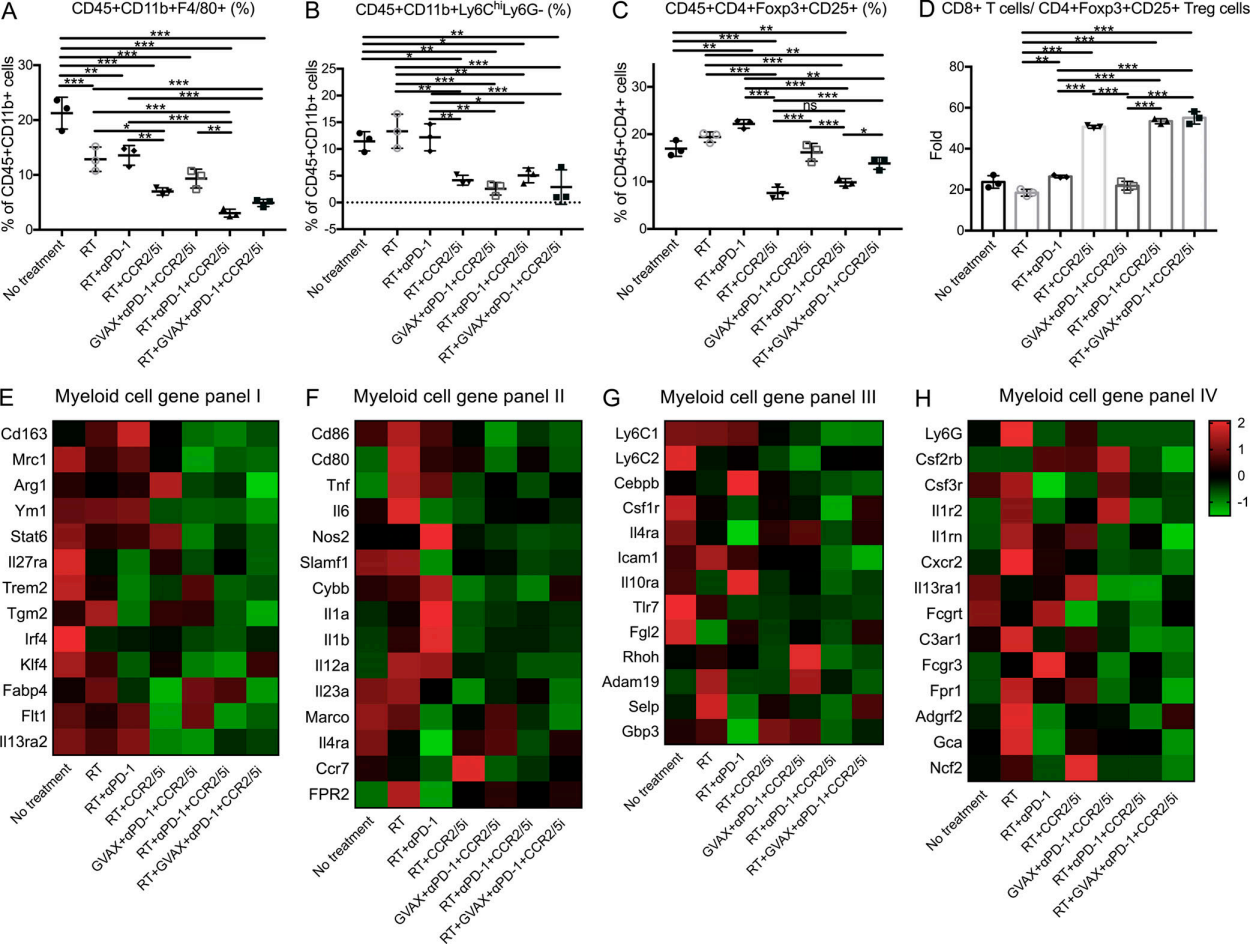
**CCR2/5 inhibitor in combination with RT and αPD-1 reverses the suppressive immune environment in a PDAC orthotopic mouse model.** Flow cytometry was performed on isolated tumor-infiltrating immune cells from dissected orthotopic tumor on day 16. **(A–D)** The number of isolated tumor-infiltrating immune cells was normalized to the tumor weight (*n* = 4–5 per group). The following were analyzed: percentage of macrophages (CD45^+^CD11b^+^F4/80^+^) among CD45^+^CD11b^+^ cells (A), M-MDSCs (CD45^+^CD11b^+^Ly6C^hi^Ly6G^−^) among CD45^+^CD11b^+^ cells (B), Tregs (CD45^+^CD4^+^CD25^+^Foxp3^+^) among CD45^+^CD4^+^ cells (C), and ratio of CD8^+^ T cells/CD4^+^CD25^+^Foxp3^+^ Tregs (D). CD11b^+^ cells were isolated from tumors of mice in different treatment groups: (1) No treatment, (2) RT, (3) RT + αPD-1, (4) RT + CCR2/5i, (5) GVAX + αPD-1 + CCR2/5i, (6) RT + αPD-1 + CCR2/5i, and (7) RT + GVAX + αPD-1 + CCR2/5i. **(E–H)** RNA was purified, amplified, and sequenced. For RNAseq results (*n* = 5 per group), heatmaps were generated to visualize the expression of signature genes whose expression was described in M2-like (E) and M1-like (F) macrophages, M-MDSCs (G), and PMN-MDSCs (H), labeled as myeloid cell gene panels I, II, III, and IV, respectively. Data represent mean ± SEM from one representative experiment of four to five mice per treatment group. For flow cytometry, the isolated immune cells from tumors of mice in the same treatment group were pooled and measured in triplicate. *, P < 0.05; **, P < 0.01; ***, P < 0.001, by one-way ANOVA.

Next, we examined the potential cellular targets of CCR2/5i in the TME of the orthotopically implanted KPC tumor model (Fig. S4 A). Tumors were harvested and digested into single-cell suspensions for flow cytometry analysis (Fig. S4 F). As shown in Fig. 5 A, groups treated with CCR2/5i had a significantly lower percentage of CD45^+^CD11b^+^F4/80^+^ TAMs among CD11b^+^ myeloid cells. Furthermore, both the tumor weight–normalized cell number and the percentage of TAMs among myeloid cells in the RT + αPD-1 + CCR2/5i group were reduced significantly compared with the RT + αPD-1 group. However, there was no difference in the density or percentage of TAMs among myeloid cells between the RT + αPD-1 + CCR2/5i and RT + GVAX + αPD-1 + CCR2/5i groups (Figs. 5 A and S4 G). Groups treated with CCR2/5i had a significantly lower percentage of M-MDSCs (CD45^+^CD11b^+^Ly6C^hi^Ly6G^−^) among myeloid cells. Both the percentage and the density of M-MDSCs in the RT + αPD-1 + CCR2/5i treatment group were significantly lower than those in the RT + αPD-1 or RT-only groups (Figs. 5 B and S4 H). The addition of GVAX to the combination of RT + αPD-1 + CCR2/5i did not further affect the M-MDSC infiltration in the tumors. By contrast, CCR2/5i did not appear to influence the density and the percentage of PMN-MDSCs in the tumors (Fig. S4 I).

In addition to macrophages and M-MDSCs, we examined Tregs (CD45^+^CD4^+^CD25^+^Foxp3^+^) in the different treatment groups (Fig. S4, J–L). RT increased Treg infiltration, and the addition of CCR2/5i decreased this RT-induced Treg infiltration (Fig. 5 C). The percentage of Tregs in the RT + αPD-1 + CCR2/5i treatment group was significantly lower than in the RT + αPD-1 and RT + GVAX + αPD-1 + CCR2/5i treatment groups (Fig. 5 C). In addition, the ratio of CD8^+^ T cells to Tregs in the RT + GVAX + αPD-1 + CCR2/5i, RT + αPD-1 + CCR2/5i, and RT + CCR2/5i groups was significantly higher compared with the no treatment control, RT-only, RT + αPD-1, and GVAX + αPD-1 + CCR2/5i groups (Fig. 5 D), suggesting that the combination of RT and CCR2/5i might tip the balance toward effector T cells and away from immunosuppressive cells in the TME.

**Figure S5.**
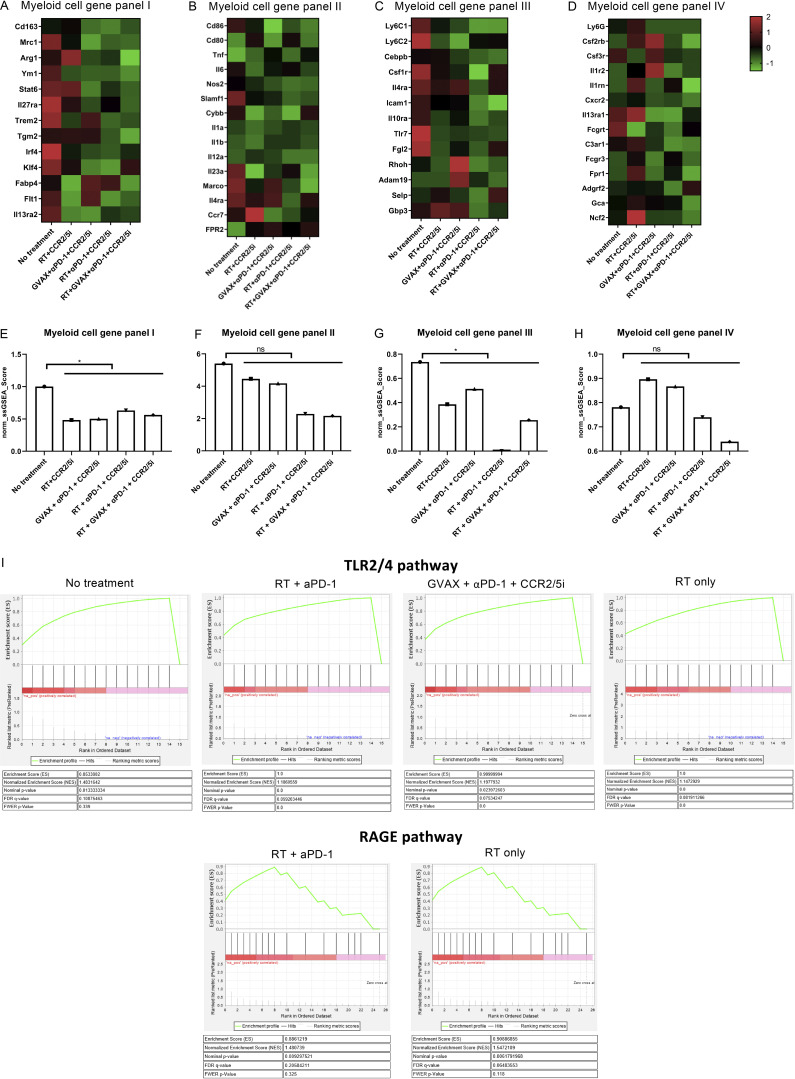
**The enrichment analysis of differentially expressed genes among treatment groups.** CD11b^+^ cells were isolated from mice using MACS sorting, RNA was purified, amplified, and sequenced. **(A–H)** Heatmaps were generated to visualize the expression of signature genes whose expression has been described in M2-like (A), M1-like macrophages (B), M-MDSCs (C), and PMN-MDSCs (D), respectively. These gene signatures in M2-like (E) and M1-like macrophages (F), and M-MDSC (G) and PMN-MDSC (H), labeled as myeloid cell gene panels I, II, III, and IV, respectively, were subjected to ssGSEA for enrichment analysis, and their expression levels (ssGSEA scores) were compared between untreated group and CCR2/5i-containing groups by *t* test. *, P < 0.05. **(I)** The differentially expressed genes in TLR2/4 and RAGE pathway were uploaded into GSEA for enrichment analysis. The h.all.v5.1.symbols.gmt [Hallmarks] gene sets database was used as the gene set collection for analysis. GSEA performed 1,000 permutations. Cutoff for significant gene sets was FDR < 25%.

Because the above human PDAC RNAseq data suggested that CCR2 and CCR5 were the main immunosuppressive signals on myeloid cells following αPD-1 therapy, we prioritized our RNAseq analysis on the CD11b^+^ myeloid cells sorted from the orthotopically implanted KPC pancreatic tumors following various treatments. Treatment combinations that included CCR2/5i (groups 4–7) were associated with lower expression of M2-like macrophage signature genes, as demonstrated in the heatmap (Fig. 5 E), compared with the untreated control group and non-CCR2/5i groups. We performed single-sample gene set enrichment analysis (ssGSEA) to compare M1-like macrophage gene signatures between the untreated group and all CCR2/5i-containing groups and found that there was no statistically significant difference (Fig. S5, B and F). In contrast, ssGSEA analysis showed a statistically significant difference in the M2-like macrophage gene signatures between the untreated group and CCR2/5i-containing groups (Fig. S5, A and E). Treatment combinations that included RT and CCR2/5i (groups 4, 6, and 7) were associated with lower expression of *Il27ra*, *Trem2*, *Tgm2*, *Irf4*, *Klf4*, and *Flt1*, suggesting that the combination of RT and CCR2/5i further suppressed M2 macrophage function. Treatment combinations that included RT and CCR2/5i were also associated with down-regulation of M-MDSC signature genes, as demonstrated in the heatmap (Fig. 5 G). ssGSEA analysis showed that there was no statistically significant difference in the PMN-MDSC–like gene signatures, but a statistically significant difference in the M-MDSC gene signatures between untreated group and CCR2/5i-containing groups (Fig. S5, C, D, G, and H). Taken together, these results suggested that, compared with the control, CCR2/5i in combination with RT and αPD-1 may have led to the reduction of immunosuppressive cells, including Tregs, M2-like TAMs, and M-MDSCs, but unlikely M1-like TAMs and PMN-MDSCs in the PDAC TME.

### Adding CCR2/5 dual inhibition to RT suppresses immunosuppressive cytokines but permits the expression of effector T cell chemokines

**Figure 6.**
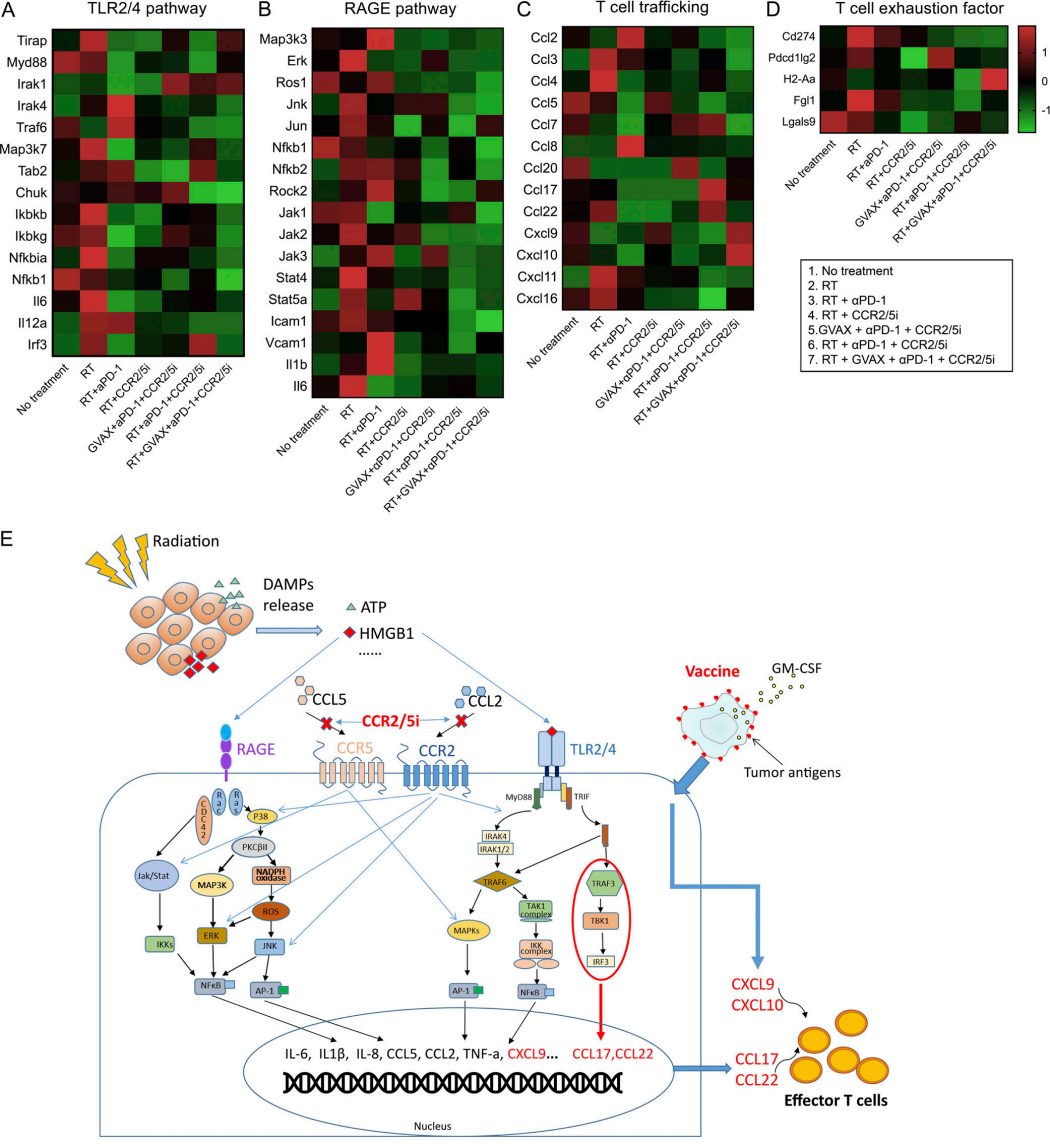
**Inhibition of CCR2 and CCR5 reverses radiation-induced TLR2/4 and RAGE signaling and permits the expression of effector T cell chemokines in αPD-1–treated pancreatic adenocarcinoma. (A–D)** CD11b^+^ cells were isolated from mice (*n* = 5 per group) using MACS sorting; RNA was purified, pooled, amplified, and sequenced; and heatmaps were generated to visualize the expression of genes associated with TLR2/4 (A), RAGE (B), T cell trafficking (C), and T cell exhaustion (D) pathways. **(E)** A working model for the mechanism of action of CCR2/5 dual inhibition when combined with RT and other immunotherapies. Damage-associated molecular pattern (DAMP) signals such as HMGB1 are released in response to RT and subsequently activate RAGE and/or TLR2/4 pathways in TAMs. The activation of the downstream signaling pathways lead to the expression of immunosuppressive cytokines/chemokines including CCL2 and CCL5. Most of these downstream pathways will be further activated by binding of CCL2/CCL5 to CCR2/5. Adding CCR2/5i to RT inhibits these signals that are shared between CCR2, CCR5, TLR2/4, and RAGE pathways; however, it does not inhibit the TRAF3–TBK1–IRF3 axis. The TRAF3–TBK1–IRF3 axis remains to be upregulated and subsequently enhances the transcription of *CCL17* and *CCL22*, two effector T cell trafficking factors, thus promoting T cell infiltration into the tumor. TRIF, TIR-domain-containing adapter-inducing interferon-β; IKK, IκB kinase.

We next analyzed the intracellular signaling pathways using the aforementioned RNAseq analysis on the sorted CD11b^+^ myeloid cells isolated from the orthotopic pancreatic tumors following various treatments. RT and chemotherapy are known to potentially induce immunogenic cell death in cancer cells. In particular, damage-associated molecular pattern signals such as HMGB1 are released by tumors in response to RT and subsequently activate the RAGE and/or TLR2/4 signaling pathways (Roses et al., 2008; Wang et al., 2019). We thus hypothesized that CCR2/5i modulates the RT-induced RAGE and TLR2/4 signaling pathways in the myeloid cells and subsequently produces T cell activation/trafficking cytokines/chemokines, leading to increased intratumoral T cell infiltration and function. Supporting this hypothesis, the RNAseq results demonstrated that RT or RT + αPD-1 activated the RAGE- and TLR2/4-mediated signaling pathways within CD11b^+^ myeloid cells (Fig. 6, A and B). These results were supported by GSEA (Table 1 and Fig. S5 I). Note that the TLR2/4 pathway was enriched in the untreated control group (false discovery rate [FDR] = 0.108), likely representing the signals induced by the spontaneous tumor cell death. Nevertheless, the TLR2/4 pathway and RAGE pathway were further enriched when RT was given (FDR = 0.075 and 0.065). The enrichment of the TLR2/4 and RAGE pathways disappeared with the addition of CCR2/5i. There was also an enrichment of the TLR2/4 pathway following GVAX + αPD-1 + CCR2/5i treatment (FDR = 0.081) as expected following treatment with GVAX (Fig. S5 I). Adding CCR2/5i to RT inhibited those “unwanted” signals (*Jak/Stat*, *ERK*, *JNK*, etc.), which are known to be regulated by CCR2 and CCR5 and thus inhibited by CCR2/5i (Bose and Cho, 2013; Sanz and Garcia-Gimeno, 2020; Wu and Yoder, 2009). Subsequently, the RAGE- and TLR2/4-induced T cell suppressive cytokines/chemokines are inhibited by CCR2/5i. Nevertheless, RT + αPD-1 + CCR2/5i increased the transcription of two effector T cell trafficking factors, *CCL17* and *CCL22*, as demonstrated in the heatmap (Fig. 6 C), likely through permitting activation of the TRAF3–TBK1–IRF3 axis. Although *CCL17* and *CCL22* also play a role in Treg trafficking, we anticipate that this role would be counteracted by the inhibitory effect of CCR2/5i on Tregs (Fig. 5 C). Thus, these results provide a clue on a new mechanism of action of RT in combination with CCR2/5i that, through an increased transcription of *CCL17* and *CCL22*, leads to increased effector T cell infiltration into the tumor and improved T cell function (Fig. 6 C). As anticipated, most T cell exhaustion factors were downregulated in the RT + αPD-1–treated groups (groups 2, 3, 5, 7) as demonstrated in the heatmap (Fig. 6 D). Taken together, this study supported the hypothesis that CCR2/CCR5 dual-antagonist licenses radiation-induced effector T cell infiltration in αPD-1–treated PDACs (Fig. 6 E).

**Table 1.** Summary of TLR2/4 and RAGE pathway enrichment

Treatment group	TLR2/4 pathway	RAGE pathway
Untreated control	Enriched (FDR = 0.108)	Not enriched
RT + GVAX + αPD-1 + CCR2/5i	Not enriched	Not enriched
RT + αPD-1 + CCR2/5i	Not enriched	Not enriched
RT + CCR2/5i	Not enriched	Not enriched
RT + αPD-1	Enriched (FDR = 0.059)	Enriched (FDR = 0.205)
RT only	Enriched (FDR = 0.075)	Enriched (FDR = 0.065)
GVAX + αPD-1 + CCR2/5i	Enriched (FDR = 0.081)	Not enriched

## Discussion

We present the first preclinical study to investigate the synergistic effects of RT and combination immunotherapy with GVAX, αPD-1, and CCR2/5i for the treatment of PDAC. In patients who had increased expression of CCR2 and CCR5 in myeloid cells after receiving combination therapy with GVAX and αPD-1, the tumor-infiltrating myeloid cells were associated with increased M2-like macrophage and M-MDSC gene signatures. In addition, patients who had increased CCR5 expression in CD4^+^ cells had increased expression of Treg gene signatures. However, the addition of CCR2/5i to GVAX + αPD-1 combination therapy did not improve survival in a mouse model of PDAC.

Because RT can induce immunogenic cell death in cancer cells, and it is well established that immune cells are crucial for the antitumor effect of RT (Demaria et al., 2015), we decided to investigate both GVAX and RT as T cell–priming agents in combination regimens that included αPD-1 and CCR2/5i in mouse models of PDAC. We found that although the addition of GVAX to RT + αPD-1 + CCR2/5i conferred early tumor control, tumor growth appeared to accelerate later in the disease course for reasons that remain to be further investigated. However, the combination therapy of RT + αPD-1 + CCR2/5i led to better survival and tumor control. This antitumor efficacy of CCR2/5 inhibition in combination of RT and αPD-1 corresponded with a decrease in the infiltration of a broad spectrum of immunosuppressive cells, including macrophages, M-MDSCs, and Tregs, into the PDAC TME. Mechanistically, this study suggests that CCR2/5 dual inhibition counteracts radiation-induced suppressive signals in myeloid cells and upregulates the effector T cell pathway possibly through CCL17 and CCL22 chemokines. However, direct evidence is still needed to demonstrate that CCL17 and CCL22 mediate the effector T cell infiltration in CCR2/5i-treated tumors. Nevertheless, the findings in this preclinical study support conducting a clinical trial of inhibiting CCR2/5 in combination with αPD-1 and RT in PDAC.

We previously published that neither RT nor RT + α-PD-1 induced CD8^+^ T cell infiltration into PDAC. In this study, we demonstrated that both RT and RT + αPD-1 treatment regimens activated RAGE- and TLR2/4-mediated signaling pathways within CD11b^+^ myeloid cells. However, adding CCR2/5i to the regimen led to inhibition of RAGE and TLR2/4 pathways, which we hypothesize led to the upregulation of the TRAF3–TBK1–IRF3 axis and the downregulation of T cell–suppressive cytokines. These changes ultimately led to increased transcription of CCL17 and CCL22, two effector T cell–trafficking factors. Therefore, this study opens a new area of radio-immunobiology where further dissection is needed for the role of this axis in inducing the CCL17 and CCL22 chemokines and in mediating effector T cell infiltration in PDAC and likely other solid tumors.

In this study, we observed that adding GVAX to RT + αPD-1 + CCR2/5i combination therapy did not improve survival in a mouse model of PDAC, which might be due to the decrease in memory T cells associated with this combination. The underlying mechanism remains to be further explored. However, the addition of GVAX to CCR2/5i + αPD-1 increased IFN-γ secretion from T cells in the liver metastatic mouse model. We did not verify whether increased T cell secretion of IFN-γ was associated with RT in combination with CCR2/5i + αPD-1, because the orthotopic model was used for treatment combinations that included RT, and very few CD8^+^ T cells could be isolated in this model.

Although our study does not support the addition of vaccine to CCR2/5i + αPD-1 therapy for PDAC in the presence of RT, our study supports the combination of RT, CCR2/5i, and αPD-1 for PDAC treatment. However, we acknowledge that there were some limitations in this study. First, the treatment effect of targeting only CCR2 or CCR5 was not tested in this study. CCR2 has been tested in multiple clinical trials and likely exerts its effects through targeting CCR2^+^ myeloid cells. CCR5, another chemokine receptor that plays a role in the infiltration of Tregs and TAMs into tumors, is another potential target for inhibition. Nevertheless, our human PDAC data allowed us to examine the relationship between the expression of CCR2 and CCR5 in different immune cell subtypes and the gene signatures of myeloid cells and Tregs. Thus, we were able to hypothesize the potential effect of CCR2 inhibition and CCR5 inhibition at the immunobiological level. As CCR5 has a unique function in Tregs, our study supported targeting CCR2 and CCR5 simultaneously in PDAC. It was suggested that the CCR5/CCL5 pathway may play a role in tumor suppression. Thus, it is possible that inhibition of CCR2 as opposed to dual CCR2/CCR5 inhibition may be superior. In future studies, an effort to distinguish the antitumor efficacy of CCR2 inhibition and CCR5 inhibition from that of dual CCR2/CCR5 inhibition is warranted.

In addition, our results were limited by the differences between the mouse model of PDAC and human PDAC, and the antitumor efficacy of the combination therapy warrants further investigation in human studies for PDAC. Nevertheless, the hemispleen liver metastatic model and orthotopic model used in this study are biologically, including immunologically, more similar to human PDAC than traditional subcutaneous tumor models. A phase 1/2 trial of combination immunotherapy with nivolumab and CCR2/5i (BMS-813160) with or without GVAX following SBRT in patients who already received chemotherapy for locally advanced PDAC (NCT03767582) is ongoing at our center.

Although combining vaccine and CCR2/5i + αPD-1 in the presence of RT did not result in a synergistic survival effect or decrease in tumor growth rate, PDACs that are primed by other mechanisms (e.g., a non–T cell-inflamed mechanism) might still benefit from combination treatment with vaccine and CCR2/5i + αPD-1. Therefore, it would be interesting to investigate whether other treatment modalities such as chemotherapy or innate immune agonists could prime PDAC for combination treatment with CCR2/5i + α-PD-1 in the presence or absence of RT.

## Materials and methods

### Cell lines

The KPC (LSL-Kras (G12D/+); LSL-Trp53 (R172H/+); Pdx-1-Cre) tumor cell line is a previously established PDAC cell line derived from a KPC mouse model in the C57Bl/6 background and cultured as previously described (Hingorani et al., 2005). B78H1-GM cells are an MHC class I–negative variant of the B16 melanoma tumor cell line, engineered to secrete GM-CSF and used to formulate whole-cell autologous GVAX vaccine. Harvested tumor-infiltrating immune cells were processed in T cell medium, which consisted of RPMI 1640 (Life Technologies) supplemented with 10% heat-inactivated FBS (Benchmark), 1% penicillin/streptomycin (Life Technologies), 1% Hepes (Life Technologies), 1% MEM Non-Essential Amino Acids Solution (Life Technologies), 1% L-glutamine (Life Technologies), and 0.05% 2-mercaptoethanol (Sigma-Aldrich).

### Mice and in vivo experiments

#### Mice

C57Bl6 mice (6–8 wk) were purchased from Harlan Laboratories and maintained in accordance with the Johns Hopkins University Institutional Animal Care and Use Committee (IACUC) guidelines. Mice considered to have reached a “survival endpoint,” including hunched posture, lethargy, dehydration, and rough hair coat, were euthanized. The IACUC mouse protocol was maintained by third-party management. The KPC liver metastatic model and pancreatic orthotopic model were described previously (Fujiwara et al., 2020; Soares et al., 2014).

#### Metastatic model

The hemispleen technique of tumor inoculation was performed on day 0. In brief, after anesthetizing the mouse, a left subcostal incision was made, and the spleen was eviscerated, clipped, and hemisected. One half of the spleen was injected with 2 × 10^5^ KPC cells resuspended in 100 μl PBS and flushed with 150 μl PBS in the same syringe. Cells were injected slowly into the exposed hemispleen, while the syringe was kept upright at all times to ensure the 150-μl PBS flush remained as free from tumor cells as possible. The splenic vessels were then clipped, and the injected hemispleen was resected to remove residual tumor cells. Following this procedure, diffuse liver metastases develop, and we previously reported that all untreated mice die in 4–6 wk. Mice were randomized to each treatment group after the surgery.

#### Orthotopic model

2 × 10^6^ KPC cells were s.c. injected into the flanks of syngeneic female C57Bl/6 mice. After 1–2 wk, the subcutaneous tumors were harvested and cut into 2-mm^3^ pieces. New syngeneic female C57Bl/6 mice, aged 8–10 wk, were anesthetized. A left subcostal incision was made in the abdomen to obtain access to the body and tail of the pancreas. A small pocket was prepared in the middle of the pancreas using microscissors, and one 2-mm^3^ piece of the subcutaneous tumor was implanted into the small pocket. The incision in the pancreas was closed with a 7-0 Prolene suture. Small Horizon Titanium Ligating Clips were carefully placed on either side of the implanted tumor (symmetrically and 5–10 mm lateral to the tumor) to be used as fiducial markers. The abdominal wall of the skin was sutured using 4-0 sutures. Mice were randomized to each treatment group after the surgery.

#### Treatment

On days 6–8 or as indicated after tumor implantation surgery, mice were anesthetized with isoflurane, and the pancreas tumors were irradiated with 8 Gy daily using the Small Animal Radiation Research Platform (Xstrahl). The isocenter was placed at the center of the fiducials. Whole-tumor-cell autologous GVAX immunotherapy was prepared using cultured KPC and GM-CSF–expressing B78H1 cells; cells were harvested, washed in PBS, combined at an equal concentration of 2 × 10^7^ cells/ml, and irradiated at 50 Gy. GVAX was administered subcutaneously in three limbs (100 μl into each limb). Anti-mouse PD-1 antibody (5 mg/kg; RMP1-14, BioXcell) or IgG (5 mg/kg; 2A3, BioXcell) were administered i.p. twice weekly. CCR2/5i (20 or 50 mg/kg; BMS-687681) was dissolved in PEG 300 (Thermo Fisher Scientific) and was administered by oral gavage twice a day. Tumor size was measured by ultrasound. Examiners were blinded to the treatment groups. Drug toxicity was assessed by mice body weight.

### Cell processing and flow cytometry

Dissected orthotopic pancreatic tumors were collected on day 16 after tumor implantation for analysis of tumor-infiltrating immune cells. Each tumor was mechanically processed through 40- and 100-mm nylon filters sequentially and brought to a volume of 20 ml in T cell medium. The cell suspensions were centrifuged at 1,500 rpm for 5 min. Cell pellets were suspended in 4 ml of Ammonium-Chloride-Potassium lysis buffer (Quality Biological) and subsequently spun at 1,500 rpm for 5 min. Cell pellets were then resuspended in 6 ml of 80% Percoll (GE Healthcare LifeSciences), overlaid with 6 ml of 40% Percoll, and centrifuged at room temperature for 25 min at 3,200 rpm without break. The leukocyte layer was removed and quenched with 30 ml of CTL medium.

After the isolation of leukocytes from the murine pancreatic tumor, leukocytes from mice in the same treatment group were pooled and stained with the Live Dead Aqua Dead Cell Kit (Invitrogen). The leukocytes were washed and blocked with mouse Fc antibody (BD Pharmingen) for 10 min on ice, followed by staining with cell surface antibodies for 30 min on ice. The cell surface antibodies used were CD45-APC Cy7 (BD Pharmingen), CD3-APC (BioLegend), CD4-APC H7 (BioLegend), CD8-PE Cy7 (BioLegend), CD25-BV421 (BioLegend), PD-1-FITC (BioLegend), CD137-APC (eBioscience), CD11b-PE TR (Life Technologies), Ly6C-PerCP Cy5.5 (eBioscience), Ly6G-V450 (BD Horizon), F4/80-PE Cy7 (eBioscience), CD44-PE (BioLegend), CD62L-APC (BioLegend), and CCR7-BV421 (BioLegend). Intracellular staining with anti-mouse forkhead box P3 (FoxP3) was performed after cell surface marker incubation. The cells were suspended in cold Fix/Perm buffer (eBioscience) and incubated for 30 min at 4°C. The cells were then washed with Perm Buffer (eBioscience). FoxP3-PE (eBioscience) antibody was added, and the cells were incubated on ice for 30 min. The cells were washed with Fix/Perm buffer, and flow cytometry was performed using CytoFLEX (Beckman Coulter). Flow data were analyzed using CytExpert software (Beckman Coulter).

### Mouse IFN-γ ELISAs

CD8-negative isolation kits (Life Technologies) were used to isolate CD8^+^ T cells from the liver and spleen of mice that underwent the hemispleen procedure for the metastatic tumor model. The isolated CD8^+^ cells from the same treatment group were pooled and cocultured with irradiated (at 50 Gy) autologous KPC tumor cells at a ratio of 5:1 (2 × 10^5^ CD8^+^ T cells: 4 × 10^4^ irradiated KPC tumor cells). The coculture was incubated for 18 h in AIM V  medium (Thermo Fisher Scientific) at 37°C. Mouse IFN-γ ELISA Ready-Set-Go (eBioscience) was then conducted with the supernatant per manufacturer’s protocol.

### RNAseq

Dissected orthotopic KPC tumors were digested into single cells and pooled from the same group. Mouse CD11b cell isolation kit (STEMCELL; positive isolation) was used to isolate tumor-infiltrating CD11b^+^ cells. TRIzol Reagent (Thermo Fisher Scientific) was used to extract total RNA from tumor-infiltrating immune cell pellets, and whole-exome RNAseq was performed by BGI. Volcano plots were generated by using the online Limma tool (volcanoplot), and genes differentially expressed were selected according to the Volcano plot analysis.

### Statistical analysis

All statistical analyses and graphing were performed using GraphPad Prism software (GraphPad Software). Kaplan–Meier curves and log-rank tests were used to estimate median survival and analyze survival outcomes between subgroups. For comparison of cell number, percentage, and cytokine expression, the mean values were evaluated using Student’s or Welch’s *t* test. One-way ANOVA was used for multiple comparisons of means. A P value <0.05 was considered statistically significant.

### Study approval

Deidentified human PDAC resection specimens were obtained from the clinical trial (NCT02451982) patients who underwent surgery at the Johns Hopkins Hospital under the Johns Hopkins Medical Institution Institutional Review Board–approved protocol (IRB00050517). All studies and maintenance of mice were conducted in accordance with the approval of the IACUC guidelines. Mice considered to have reached a survival endpoint, including hunched posture, lethargy, dehydration, and rough hair coat, were euthanized.

### Data availability

RNAseq data are deposited at GEO with the accession number GSE197613. They are also available as Data S1 for results in Fig. 1 and Data S2 for results in Figs. 5 and 6.

### Online supplemental material

Fig. S1 shows that immunosuppressive cell infiltration was associated with CCR2 and CCR5 expression in human PDAC tissue. Fig. S2 shows that adding GVAX to dual antagonism of CCR2 and CCR5 in combination with αPD-1 does not significantly enhance survival in a murine PDAC model. Fig. S3 shows the treatment schema and tumor growth curve in different treatment groups. Fig. S4 shows that CCR2/5 dual antagonist, in combination with RT and anti–PD-1 therapy, enhanced effector memory T cell infiltration and reversed the suppressive immune cell environment. Fig. S5 shows the enrichment analysis of differentially expressed genes among treatment groups. Data S1 shows RNAseq results in Fig. 1. Data S2 shows RNAseq results in Figs. 5 and 6.

## Supplementary Material

Data S1 shows RNAseq results in Fig. 1.

Data S2 shows RNAseq results in Figs. 5 and 6.
